# Enhancer of Zeste Homolog 2 (EZH2): From Glioblastoma Biology to Potential Epigenetic Therapy

**DOI:** 10.3390/ijms27177749

**Published:** 2026-08-29

**Authors:** Jan Grzegorzewski, Maria Lindner, Dagmara Lisińska, Aleksandra Majchrzak-Celińska

**Affiliations:** 1 Poznan University of Medical Sciences, Doctoral School, Bukowska 70, 60-812 Poznań, Poland; j.grzegorzewski2001@gmail.com; 2 Poznan University of Medical Sciences, Department of Pharmaceutical Biochemistry, Rokietnicka 3, 60-806 Poznań, Poland; lindner.maria02@gmail.com (M.L.); lisinskad@gmail.com (D.L.); 3 Poznan University of Medical Sciences, The Student Scientific Society Biomolekularni, 60-806 Poznań, Poland

**Keywords:** EZH2, histone methylation, glioblastoma, GBM, HOTAIR, AGAP2-AS1, epigenetic therapy

## Abstract

Epigenetic dysregulation is a hallmark of glioblastoma (GBM) pathogenesis, with histone methylation playing a central role in chromatin remodeling and gene expression regulation. Enhancer of zeste homolog 2 (EZH2) is a key epigenetic regulator responsible for histone methylation. It primarily functions as a transcriptional repressor and regulates signaling pathways associated with tumor progression. Importantly, EZH2 is frequently recruited to long non-coding RNA (lncRNA) scaffolds, including HOTAIR and AGAP2-AS1, thereby enabling coordinated gene silencing by establishing repressive chromatin states. Although *EZH2* expression is not consistently associated with overall survival in GBM, EZH2 remains a promising therapeutic target due to its roles in stemness and treatment resistance. This review summarizes current knowledge of EZH2 functions in normal and GBM cells, highlighting its complex functions and therapeutic potential. We also discuss the current status and limitations of EZH2-targeting therapies, including challenges related to blood–brain barrier penetration and clinical translation. Although a combination of EZH2 inhibitors and other epigenetic inhibitors may be beneficial in selected molecular contexts, its rationale depends on the underlying regulatory network. Future therapeutic development will require biomarker-driven patient stratification and precision medicine approaches to maximize clinical benefit in GBM.

## 1. Introduction

Representing the most prevalent malignant primary brain tumor, glioblastoma (GBM) accounts for 14.5% of all tumors arising within the central nervous system (CNS) and nearly half (48.6%) of all malignant CNS neoplasms [1,2,3]. It is classified by the World Health Organization (WHO) as a grade 4 glioma, the highest level of malignancy [1]. Although its annual incidence is relatively low, at approximately 3–4 cases per 100,000 individuals, the risk of developing GBM increases markedly with advancing age and is more frequently observed in males. Reflecting this age-dependent distribution, the median age at diagnosis is 65 years [4]. Moreover, GBM is widely recognized as one of the most aggressive human cancers, posing substantial challenges for clinical management and therapeutic intervention [1]. The non-specific nature of presenting symptoms, such as headaches, ataxia, dizziness, vision, and recurrent syncope, often complicates diagnosis and may contribute to delayed initiation of treatment [5].

### 1.1. Current Standard of Care

The current standard of care for patients with GBM consists of maximal safe surgical resection followed by concomitant radiotherapy and temozolomide (TMZ), with subsequent adjuvant TMZ administered for six monthly cycles [3,6]. In addition, the integration of tumor treating fields (TTFs) into the therapeutic strategy has been associated with improved survival and clinical outcomes in patients with newly diagnosed disease [7]. Despite these advances, GBM continues to be characterized by a poor prognosis owing to its diffuse infiltrative growth pattern, marked therapeutic resistance, and the tumor’s anatomically constrained location within the brain [8]. Over the past three decades, the survival rates for individuals diagnosed with GBM have experienced only limited improvement [9]. Following surgical intervention, overall survival rarely exceeds two years, while the five-year survival rate remains approximately 7.1% [4]. The median overall survival for GBM patients is around 15 months [10].

### 1.2. Resistance Mechanisms

The persistence of tumor progression, therapeutic resistance, and recurrence in GBM are closely linked to its pronounced intratumoral heterogeneity and cellular plasticity [11,12,13]. These characteristics are largely driven and maintained by glioma stem cells (GSCs), a specialized subpopulation endowed with self-renewal capacity, tumor-initiating potential, genomic instability, and the ability to generate diverse cellular states, thereby promoting tumor heterogeneity and adaptive evolution [14,15]. Beyond their role in tumor maintenance, GSCs contribute to therapeutic resistance through multiple mechanisms, including evasion of apoptosis, modulation of the tumor microenvironment (TME), induction of angiogenesis, immunosuppression, and promotion of radio- and chemoresistance [16,17,18]. However, accumulating evidence indicates that treatment failure results from the coordinated interplay of several resistance mechanisms rather than GSC activity alone. Enhanced DNA repair capacity, metabolic reprogramming, and a permissive TME, together with the highly infiltrative nature of GBM and the protective function of the blood–brain barrier (BBB), collectively limit therapeutic efficacy and contribute to the extraordinary resilience of this malignancy against conventional treatment strategies [13,18,19,20,21].

### 1.3. Genetics and Epigenetics of GBM

The molecular landscape of GBM is shaped by a combination of genetic and epigenetic alterations. GBM is characterized by the absence of mutations in the isocitrate dehydrogenase (*IDH)* gene (*IDH-*wildtype) and commonly exhibits molecular abnormalities involving the *TERT* promoter, Epidermal Growth Factor Receptor (*EGFR*) amplification, and characteristic chromosomal aberrations, including chromosome 7 gain and chromosome 10 loss [22,23]. In addition, disruption of tumor suppressor pathways plays a critical role in tumor development. *TP53* mutations occur in approximately 25% of GBMs and in more than 60% of astrocytoma, *IDH*-mutant, CNS WHO grade 4, leading to impaired p53 function, genomic instability, and the accumulation of further oncogenic events that facilitate malignant transformation [24,25]. *PTEN* inactivation has also been implicated in promoting tumor progression through changes in protein stability and cellular localization [26]. It is important to note that *IDH-*mutant gliomas, including astrocytomas and oligodendrogliomas, represent a biologically distinct group of tumors that generally exhibit slower disease progression and are associated with a more favorable clinical outcome than *IDH*-wildtype GBM [27].

Growing evidence highlights the importance of epigenetic dysregulation in GBM, which contributes to tumor initiation, progression, and therapy resistance [28,29,30,31,32,33]. Epigenetics refers to reversible changes in gene expression that occur without modifications in the DNA sequence, providing an additional regulatory layer that shapes cellular identity and tumor plasticity in cancer [28,30,34].

DNA methylation is one of the best-characterized epigenetic alterations in GBM, contributing to both tumor progression and therapeutic response [31,35,36,37,38]. Mediated by DNA methyltransferases, it primarily occurs in CpG-rich promoter regions, where aberrant hypermethylation can silence tumor suppressor and DNA repair genes [35,36,39,40]. A clinically relevant example is O-6-methylguanine-DNA methyltransferase (*MGMT*) promoter methylation, which predicts improved response to TMZ due to reduced DNA repair capacity for alkylating damage [35,39,41]. In addition, the glioma CpG island methylator phenotype (G-CIMP), which is strongly associated with *IDH1/2* mutations, defines a distinct glioma subtype characterized by widespread epigenetic reprogramming and altered differentiation states [28,34].

GBM biology is additionally driven by histone modifications that regulate chromatin accessibility and gene expression. Histone acetylation, controlled by histone acetyltransferases (HATs) and histone deacetylases (HDACs), influences transcriptional activity, whereas histone methylation can either activate or repress gene expression depending on the modified residue and methylation state. Notably, H3K4 methylation is associated with transcriptional activation, whereas H3K9me2/3 and H3K27me3 are generally linked to transcriptional repression, while H3K36me3 correlates with transcriptional elongation [28,31,34,42]. Together, these modifications contribute to the transcriptional plasticity that characterizes GBM.

**Figure 1:**
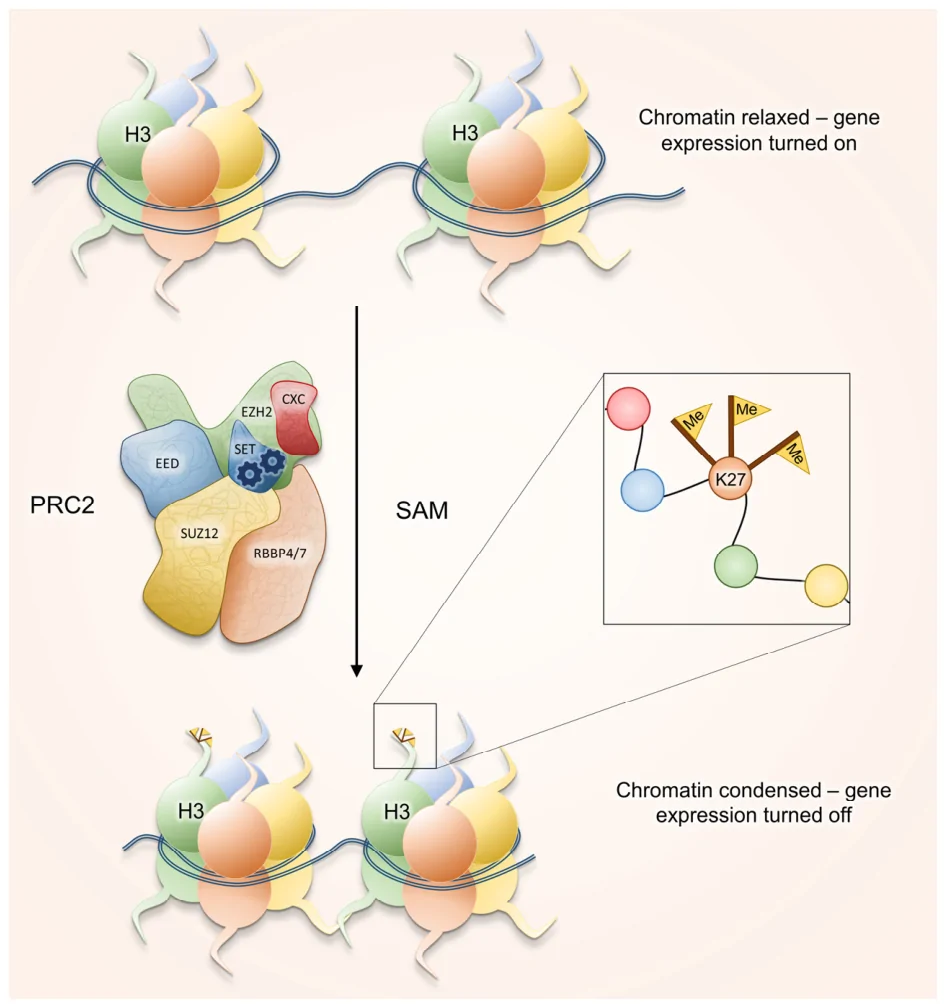
Primary mechanism of action of EZH2. EZH2 catalyzes mono-, di-, and trimethylation of H3K27, ultimately generating H3K27me3, one of the best-characterized repressive histone marks. Me—methyl group, EZH2—enhancer of zeste homolog 2, PRC2—Polycomb Repressive Complex 2, SAM—S-adenosyl-L-methionine.

The regulation of histone methylation depends on a dynamic system of “writers”, “readers”, and “erasers”, which establish, recognize, and remove methylation marks, respectively, thereby coordinating chromatin states and gene expression [28,34]. Within this framework, particular attention has been directed toward enhancer of zeste homolog 2 (EZH2), which represents crucial histone methylation regulator [43]. As the catalytic subunit of the Polycomb Repressive Complex 2 (PRC2), EZH2 functions as a histone methylation “writer”, mediating the deposition of the repressive H3K27me3 mark, which leads to chromatin compaction and transcriptional silencing [44] (Figure 1).

In GBM, EZH2 is frequently dysregulated and converges on epigenetic programs that support GSC maintenance, tumor progression, and therapeutic resistance [45,46]. EZH2 has been implicated in the promotion of GSC self-renewal and stemness, partly through epigenetic repression of tumor suppressor programs and modulation of oncogenic signaling networks, including STAT3-, AKT- and c-Myc-driven pathways [46,47].

Given its central role in shaping oncogenic chromatin, EZH2 has emerged as a promising therapeutic target in GBM. Pharmacological inhibitors of this enzyme have demonstrated antitumor activity in preclinical GBM models [43,45,48,49,50]. However, these effects are often partial and context-dependent, reflecting the complexity and redundancy of epigenetic regulatory networks in GBM [45]. A deeper understanding of EZH2-driven epigenetic regulation may provide important insights into GBM biology and facilitate the development of more effective epigenetic therapeutic strategies. In this review, we discuss the molecular functions of EZH2 in GBM, its downstream targets and signaling networks, and recent advances in the development of therapeutic inhibitors targeting this enzyme. As this is a narrative review, a formal systematic search strategy was not employed. Relevant literature was identified primarily through PubMed/MEDLINE searches using combinations of terms including “EZH2”, “histone methylation”, “glioblastoma”, “GBM”, “HOTAIR”, “AGAP2-AS1”, “epigenetic therapy”, and related keywords. Articles published up to August 2026 were considered.

Throughout this review, disease designations are reported according to the terminology used in the original publications. Because many studies were conducted before integrated molecular classification became routine, historical terms such as ‘glioblastoma’ are not retrospectively equated with GBM, *IDH*-wildtype, unless sufficient molecular information is available.

Additionally, to evaluate the clinical relevance of EZH2 in GBM, publicly available TCGA-GBM and TCGA-GBMLGG datasets were interrogated using the GlioVis platform, and the resulting expression and survival analyses were provided [51].

## 2. EZH2: Structure and Function

EZH2 is a histone lysine methyltransferase and the catalytic subunit of PRC2, one of the most important epigenetic complexes involved in transcriptional repression [44,52,53]. *EZH2* was originally identified as the mammalian homolog of the *Drosophila Enhancer of zeste* gene, encoding a member of the Polycomb group (PcG) proteins responsible for maintaining stable silencing of developmental genes during embryogenesis [52]. Unlike transcription factors, which directly recognize DNA sequences, EZH2 regulates gene expression by modifying chromatin structure through histone methylation. Its activity is essential for embryonic development, maintenance of stem cell identity, lineage specification, and tissue homeostasis, while dysregulation of EZH2 has been associated with numerous human diseases, particularly hematological malignancies and solid tumors [44,53,54,55].

### 2.1. Structure

Structurally, EZH2 is a multidomain protein composed of several highly conserved regions that coordinate its catalytic and regulatory functions. The C-terminal SET domain constitutes the enzyme’s catalytic core and transfers methyl groups to lysine residues using S-adenosyl-L-methionine (SAM) as the methyl donor [55,56]. Adjacent to the SET domain is the CXC domain, which contributes to nucleosome recognition and stabilization of the catalytic center. The N-terminal region contains additional conserved motifs involved in protein–protein interactions and assembly of the PRC2 complex [57]. EZH2 is catalytically inactive as an isolated protein and requires incorporation into the PRC2 complex together with SUZ12, EED, RBBP4/7 and accessory proteins such as AEBP2 and JARID2. These interactions stabilize the active conformation of EZH2 and are essential for efficient methyltransferase activity on nucleosomal substrates [52,57,58].

### 2.2. Function

The biochemical function of EZH2 is based on SAM-dependent methyl transfer to lysine 27 of histone H3. EZH2 sequentially catalyzes mono-, di-, and trimethylation of H3K27, ultimately generating H3K27me3, one of the best-characterized repressive histone marks in mammalian chromatin [44,52,56]. Deposition of H3K27me3 promotes recruitment of Polycomb Repressive Complex 1 (PRC1), which catalyzes ubiquitination of histone H2A and induces chromatin compaction, thereby causing stable transcriptional silencing [53,59]. Unlike histone acetylation or phosphorylation, Polycomb-mediated repression can persist through multiple rounds of cell division, providing an epigenetic memory mechanism that preserves cell identity during development and differentiation [52,53,60].

Although H3K27 trimethylation represents the principal enzymatic activity of EZH2, its biological function extends beyond histone modification. Increasing evidence indicates that EZH2 also methylates several non-histone substrates and participates in transcriptional regulation through mechanisms independent of its methyltransferase activity [47]. Furthermore, EZH2 interacts with numerous transcription factors, chromatin remodelers, and signaling pathways, allowing it to integrate extracellular stimuli with epigenetic regulation of gene expression [55]. The functional outcome of EZH2 activity therefore depends not only on its catalytic function but also on the composition of PRC2, chromatin accessibility, and the transcriptional landscape of a particular cell type.

One of the major biological functions of EZH2 is the maintenance of stem cell identity and suppression of premature differentiation. During embryonic development, PRC2 represses developmental regulators that are not required in a particular lineage while preserving the capacity of stem cells to differentiate into multiple cell types [44,52,61]. As differentiation proceeds, many PRC2 target genes lose H3K27me3, allowing activation of lineage-specific transcriptional programs. Consequently, abnormal persistence of EZH2 activity can prevent differentiation and maintain cells in an immature, highly proliferative state, a hallmark observed in many cancers [53,54,59].

Aberrant expression or activation of EZH2 has been reported in numerous malignancies, including prostate cancer, breast cancer, lymphoma, melanoma, and GBM [29,52,55,59,60].

## 3. Dysregulation of EZH2 in GBM

**Figure 2:**
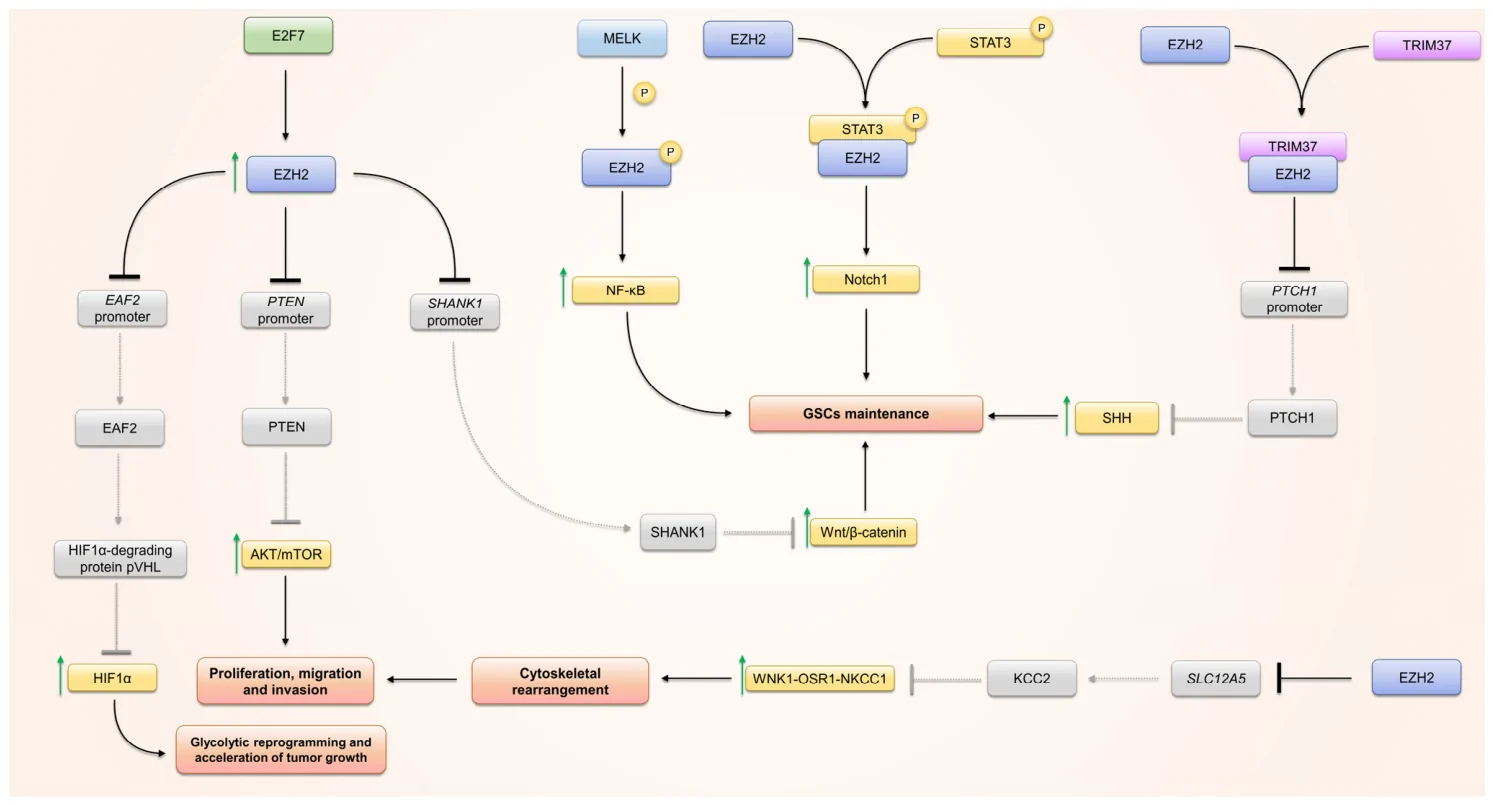
EZH2-driven signaling network promoting glioblastoma stem cell (GSC) maintenance, tumor progression, and cytoskeletal remodeling. EZH2 regulates multiple oncogenic pathways through both canonical histone methyltransferase activity and non-canonical signaling functions. E2F7 enhances EZH2 expression, which subsequently represses the *EAF2*, *PTEN,* and *SHANK1* promoters through H3K27 methylation. Repression of EAF2 decreases stabilization of the HIF1α-degrading protein pVHL, resulting in HIF1α accumulation and promotion of glycolytic reprogramming and tumor growth. Silencing of *PTEN* activates AKT/mTOR signaling, leading to enhanced tumor cell proliferation, migration, and invasion. EZH2-mediated repression of *SHANK1* reduces SHANK1 expression, thereby relieving inhibition of Wnt/β-catenin signaling and supporting GSC maintenance. Phosphorylation of EZH2 by MELK promotes NF-κB activation, which further contributes to maintenance of the GSC phenotype. In a non-canonical mechanism, phosphorylated EZH2 interacts with STAT3 to enhance Notch1 signaling, thereby sustaining GSC self-renewal. EZH2 also cooperates with TRIM37 to repress the *PTCH1* promoter, resulting in decreased PTCH1 expression and activation of SHH signaling, which further promotes GSC maintenance. In addition, EZH2 represses *SLC12A5* expression through promoter methylation, leading to reduced KCC2 levels and activation of the WNK1-OSR1-NKCC1 pathway, which drives cytoskeletal rearrangement and contributes to invasive behavior. Collectively, these interconnected EZH2-dependent pathways regulate stemness, metabolic adaptation, proliferation, invasion, and cytoskeletal dynamics in GBM. EZH2, enhancer of zeste homolog 2; GSCs, glioblastoma stem cells; SHH, Sonic Hedgehog; PTCH1, patched 1; MELK, maternal embryonic leucine zipper kinase; NF-κB, nuclear factor κB; KCC2, potassium-chloride cotransporter 2; NKCC1, sodium-potassium-chloride cotransporter 1.

EZH2 acts as a master convergence hub for disparate oncogenic signals in GBM. It is consistently overexpressed in gliomas, with the highest levels observed in GBM, supporting its involvement in aggressive tumor biology, immune evasion, and altered signaling [50,62,63,64]. In GBM, increased EZH2 activity leads to the widespread accumulation of H3K27me3 and the silencing of tumor suppressor genes, differentiation-associated genes, and regulators of apoptosis, thereby promoting uncontrolled proliferation, invasion, immune evasion, and therapeutic resistance [55,57]. In GBM, EZH2 is also highly expressed in GSCs, where it supports self-renewal, maintains stemness-associated transcriptional programs, and suppresses neural differentiation pathways [29,60]. Pharmacological inhibition or genetic depletion of EZH2 reduces proliferation, impairs tumor formation, promotes differentiation, and sensitizes GBM cells to chemotherapy and radiotherapy, highlighting its potential as a therapeutic target [29,59]. The simplified EZH2-driven signaling network in GSCs is presented in Figure 2.

### 3.1. Canonical Epigenetic Silencing

A substantial body of work converges on EZH2’s role in epigenetically silencing *PTEN* to activate AKT/mTOR signaling, though the upstream recruiting mechanism varies by context. E2F7 transcriptionally upregulates EZH2, which deposits H3K27me3 at the *PTEN* promoter to activate AKT/mTOR and drive proliferation, migration, and invasion; EZH2 restoration in E2F7-silenced cells rescued this phenotype [65]. Furthermore, EZH2-mediated repression of SHANK1 unleashes Wnt/β-catenin signaling to sustain GSC self-renewal and tumorigenicity, with SHANK1 restoration suppressing both self-renewal and β-catenin target gene expression [66]. Collectively, EZH2 acts through multiple distinct upstream scaffolds to engage the PTEN/AKT and Wnt/β-catenin axes, converging on a shared growth-promoting output.

### 3.2. Metabolic and Microenvironment Reprogramming

EZH2 also reprograms GBM metabolism toward aerobic glycolysis. By depositing H3K27me3 at the promoter of ELL-associated factor 2 (*EAF2*), a stabilizer of the HIF1α-degrading protein pVHL, EZH2 silences *EAF2* and thereby stabilizes HIF1α signaling. *EZH2/EAF2* expression is inversely correlated, and EZH2 overexpression increases glucose uptake, glycolytic enzyme activity, and lactate production while suppressing mitochondrial respiration. These effects are reversed by knockdown of EZH2 or HIF1α. This establishes an EZH2–EAF2–HIF1α axis that drives glycolytic reprogramming and accelerates tumor growth in vivo [67]. EZH2 also promotes GBM progression indirectly, by acting inside tumor-associated immune cells rather than the tumor cells themselves. Single-cell RNA sequencing of eight GBM tumors identified an EZH2-high, M2-like macrophage subset (Macrophage.3) that engages tumor cells via SPP1–CD44 and CD74–MIF signaling. It is enriched in GBM relative to low-grade glioma, and its gene signature predicts worse survival. Overexpressing EZH2 in macrophages and co-culturing them with glioma cells directly boosted tumor cell proliferation, establishing a causal role for macrophage-intrinsic EZH2 in GBM progression [68].

### 3.3. GSC Maintenance

EZH2 drives GSC maintenance through several distinct signaling axes, including noncanonical, phosphorylation-dependent signaling. In GSCs, MELK phosphorylates EZH2, enabling it to bind and methylate NF-κB independent of EZH2’s classical H3K27 methyltransferase activity. This MELK-EZH2-NF-κB axis is enriched in GSCs and high-grade gliomas, correlates with proliferation and poor patient survival, and is required to sustain stemness gene expression and self-renewal. Disrupting any node of this axis, through EZH2/NF-κB knockdown or pharmacologic inhibition (DZNep, ACHP), impairs GSC colony formation, reduces tumor growth in vivo, and pushes tumors toward a differentiated, less proliferative phenotype, identifying EZH2 phosphorylation and NF-κB methylation as a distinct, PRC2-independent mechanism through which EZH2 sustains GSC identity [62]. EZH2 also activates Notch1 signaling through STAT3, with EZH2 levels higher in GSCs than in bulk tumor cells. EZH2 knockdown reduces CD133/Nestin expression and impairs self-renewal and invasion, while the EZH2 inhibitor GSK126 reproduces these effects and suppresses tumor growth in vivo, establishing an EZH2–STAT3–Notch1 axis [69]. A third axis operates through TRIM37, which scaffolds EZH2 to repress PTCH1, and thereby disinhibits SHH signaling, supporting GSCs proliferation, spheroid formation, and SOX2 expression. EZH2 inhibition reverses this phenotype, and EZH2 restoration counteracts TRIM37 knockdown, confirming EZH2 as the operative mediator of the TRIM37-EZH2-PTCH1-SHH axis [70].

### 3.4. Cytoskeletal Rearrangement and Cell Motility

EZH2 overexpression enhances glioma cell proliferation, invasion, and migration. It exerts these effects in part by epigenetically silencing *SLC12A5*, the gene encoding the chloride transporter KCC2. EZH2 overexpression is associated with increased promoter DNA hypermethylation at *SLC12A5*, accompanied by reduced KCC2 expression [71]. As EZH2 itself has no DNA methyltransferase activity, this hypermethylation is presumed to arise indirectly—PRC2 has been shown, in other systems, to recruit DNA methyltransferases (DNMTs) to H3K27me3-marked loci [72], though this specific intermediate step (PRC2/DNMT recruitment at the *SLC12A5* locus) was not directly tested by Zhang et al. This establishes an inverse correlation between *EZH2* and *SLC12A5* expression that is also linked to prognosis. Loss of KCC2 disrupts intracellular chloride regulation and activates the WNK1-OSR1-NKCC1 signaling cascade. Downstream, phosphorylated NKCC1 interacts with ERM (ezrin–radixin–moesin) proteins to drive cytoskeletal rearrangement, promoting glioma cell migration [71].

### 3.5. Modulation Involving ncRNAs

The expression and catalytic activity of EZH2 do not function in isolation—rather, they are tightly embedded within a complex, reciprocal web of non-coding RNAs (ncRNAs), including microRNAs (miRNAs) and lncRNAs [73]. This ncRNA-EZH2 network operates primarily through two distinct mechanistic archetypes. In the first archetype, EZH2 acts as an upstream epigenetic repressor. As the catalytic subunit of PRC2, EZH2 deposits only the histone mark H3K27me3—it does not itself methylate DNA. DNA methylation at these loci is a separate, secondary event: PRC2 physically recruits DNMTs to H3K27me3-marked regions, and it is the DNMTs, not EZH2, that catalyze promoter hypermethylation there [72]. Through this two-step, PRC2-mediated mechanism, EZH2 indirectly drives silencing of tumor-suppressive miRNAs or lncRNAs, thereby derepressing downstream oncogenes that govern invasion and stemness [64,74]. In the second, reciprocal archetype, aberrant lncRNAs act as competitive endogenous RNAs or “sponges.” These transcripts selectively sequester tumor-suppressive miRNAs, preventing them from downregulating EZH2, which indirectly drives the accumulation of EZH2 protein and amplifies its oncogenic output [75,76]. These diverse, multi-layered regulatory circuits are summarized in Table 1 below.

**Figure 3:**
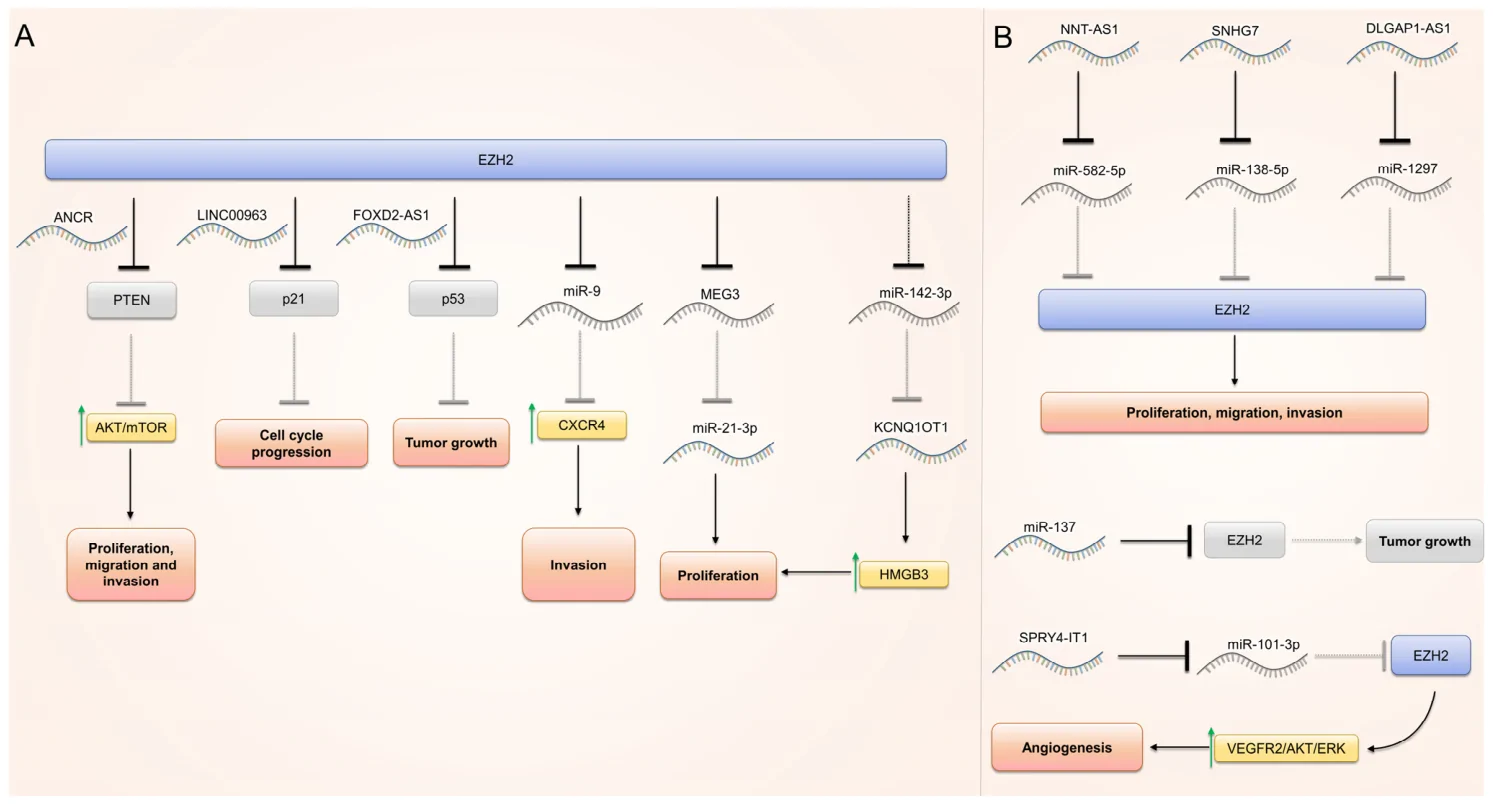
Regulatory interactions between EZH2, long non-coding RNAs (lncRNAs), and microRNAs (miRNAs) in GBM progression. (**A**) EZH2 promotes GBM malignancy through direct epigenetic repression of tumor suppressor genes and regulatory non-coding RNAs. EZH2-mediated silencing of PTEN, facilitated by the lncRNA ANCR, activates the AKT/mTOR pathway, resulting in enhanced proliferation, migration, and invasion. LINC00963 recruits EZH2 to repress p21, thereby promoting cell-cycle progression, whereas FOXD2-AS1 suppresses p53 expression through EZH2-dependent mechanisms, stimulating tumor growth. EZH2 also represses miR-9, leading to increased CXCR4 expression and enhanced invasive potential. In addition, EZH2 suppresses the tumor suppressor lncRNA MEG3, resulting in reduced miR-21-3p activity and increased cellular proliferation. Repression of miR-142-3p further promotes expression of the oncogenic factor HMGBM3, contributing to glioma cell proliferation. (**B**) Several lncRNAs regulate EZH2 expression through competing endogenous RNA (ceRNA)-like mechanisms. NNT-AS1, SNHG7, and DLGAP1-AS1 promote EZH2 expression by sequestering miR-582-5p, miR-138-5p, and miR-1297, respectively, thereby enhancing GBM cell proliferation, migration, and invasion. Conversely, miR-137 directly inhibits EZH2 expression and suppresses tumor growth. The SPRY4-IT1/miR-101-3p/EZH2 regulatory axis contributes to angiogenesis through activation of the VEGFR2/AKT/ERK signaling pathway. Collectively, these findings highlight the central role of EZH2 as an epigenetic effector integrated within lncRNA-miRNA regulatory networks that drive GBM progression, invasion, proliferation, and vascularization. ANCR, anti-differentiation non-coding RNA; AKT, protein kinase B; CXCR4, C-X-C motif chemokine receptor 4; DLGAP1-AS1, DLGAP1 antisense RNA 1; EZH2, enhancer of zeste homolog 2; FOXD2-AS1, FOXD2 antisense RNA 1; HMGBM3, high mobility group box 3; KCNQ1OT1, KCNQ1 opposite strand/antisense transcript 1; lncRNA, long non-coding RNA; LINC00963, long intergenic non-protein coding RNA 963; MEG3, maternally expressed gene 3; miRNA (miR), microRNA; NNT-AS1, NNT antisense RNA 1; PTEN, phosphatase and tensin homolog; SNHG7, small nucleolar RNA host gene 7; SPRY4-IT1, SPRY4 intronic transcript 1; VEGFR2, vascular endothelial growth factor receptor 2; AKT/ERK, protein kinase B/extracellular signal-regulated kinase.

The discovery of these layered, multi-step ncRNA-EZH2 circuits suggests that disrupting these specific sponge networks could provide a powerful avenue for combination therapy [82]. Collectively, these observations demonstrate that EZH2 sits at a critical convergence hub for ncRNA signaling. Targeting EZH2 directly or disrupting the specific lncRNA/miRNA nodes that feed into it offers a promising multi-pronged strategy to combat therapeutic resistance and tumor progression in GBM [77,79]. The simplified interactions between EZH2 and ncRNAs are depicted in Figure 3.

### 3.6. Post-Translational Control and Protein Stability

While ncRNA networks govern EZH2 predominantly at the transcriptional and post-transcriptional levels, a distinct layer of regulation operates entirely at the level of protein stability. EZH2 is a key mediator of the oncogenic effects of PSMC2 (Proteasome 26S Subunit, ATPase 2) in glioma, a protein canonically known as a proteasomal subunit but which also acts as an oncogene in several cancer types. PSMC2 expression increases with WHO tumor grade, independently predicts poor prognosis, and is elevated in glioma cell lines relative to normal astrocytes; EZH2 expression correlates positively with PSMC2 and shows the same grade-dependent increase, and PSMC2 manipulation correspondingly alters EZH2 protein levels. EZH2 knockdown alone reduces proliferation, invasion, and migration and reverses EMT (epithelial to mesenchymal transition) marker expression, paralleling PSMC2 loss-of-function effects, while EZH2 overexpression partially rescues PSMC2-knockdown phenotypes, confirming EZH2 as a major downstream effector. The precise mechanism, whether PSMC2 regulates EZH2 stability via ubiquitin-proteasome degradation, remains untested, but the convergent expression, interaction, and rescue data support PSMC2–EZH2 as a novel regulatory axis [84].

A second stability-regulating mechanism involves PRMT6, which is highly expressed in invasive, mesenchymal-subtype GBM and is associated with poor prognosis. PRMT6 protects EZH2 from degradation by epigenetically silencing the E3 ubiquitin ligase TRAF6 (via the histone mark H3R2me2a), thereby reducing TRAF6-mediated ubiquitination of EZH2 and extending its half-life. This PRMT6-TRAF6-EZH2 axis drives GBM invasion and migration in vitro and in vivo and is blocked by PRMT6 inhibitor EPZ020411. PRMT6 thus joins a growing list of enzymes (PRMT1, CARM1, PRMT5) that stabilize EZH2 post-translationally, establishing that EZH2’s oncogenic role in glioma is controlled not only transcriptionally but also through protein stability, offering a therapeutic angle distinct from direct EZH2 inhibition [85].

### 3.7. Non-Canonical Activity of EZH2

Although EZH2 has traditionally been regarded as a chromatin-associated histone methyltransferase, increasing evidence indicates that several of its non-canonical functions are mediated independently of H3K27 trimethylation. Rather than acting as a bona fide RNA-binding protein, EZH2 appears to regulate mRNA stability indirectly through interactions with non-coding RNAs, RNA-associated proteins, and microRNA regulatory networks. The EZH2-mediated stabilization of Twist1 mRNA therefore most likely reflects post-transcriptional regulation secondary to miRNA repression (e.g., miR-206) rather than direct RNA binding by EZH2 itself. Notably, EZH2 stabilizes Twist1 mRNA by suppressing miR-206, without affecting Twist1 promoter activity or pre-mRNA levels, through a mechanism entirely independent of EZH2’s canonical methyltransferase function. EZH2 knockdown increases TMZ sensitivity and reduces EMT markers, and re-expressing Twist1 fully rescues these effects, while miR-206 inhibition restores Twist1 mRNA stability, demonstrating that EZH2 can drive malignancy through post-transcriptional mRNA stabilization rather than chromatin silencing alone [63,86,87,88]. These findings broaden the functional repertoire of EZH2 beyond chromatin remodeling and emphasize that catalytic inhibition of H3K27 methylation may not fully suppress all oncogenic activities of EZH2.

### 3.8. EGFR–mTOR Signaling and EZH2 Regulation

EGFR-mTOR signaling acts as an upstream regulator of EZH2 in GBM, revealing that EZH2’s contribution to H3K27me3 is only half of a two-part mechanism. *EGFR* mutation/amplification, present in ~60% of GBMs, correlates with elevated H3K27me3 in patient tissue, an effect traced to the two parallel arms of mTOR signaling that control H3K27me3 through distinct mechanisms. mTORC1 (via Raptor) controls EZH2 at the protein level: Raptor knockdown sharply reduces EZH2 protein without affecting its mRNA, while rapamycin (mTORC1 inhibition) reduces EZH2 dose-dependently. Re-expression of EZH2 cDNA rescues the resulting H3K27me3 loss, identifying EZH2 translation, not transcription, as the mTORC1-dependent step. By contrast, mTORC2 (via Rictor) leaves EZH2 levels untouched and instead controls EZH2’s catalytic activity. Rictor knockdown depletes intracellular SAM, the methyl donor EZH2 requires for methyltransferase function, and exogenous SAM supplementation restores H3K27me3 despite Rictor loss. Functionally, this EZH2/SAM-driven H3K27me3 silences the tumor suppressor CDKN1A (p21), and pharmacologically restoring H3K27me3 with the demethylase inhibitor GSKJ4 reverses the growth-suppressive effects of either Raptor or Rictor knockdown, confirming H3K27me3 as the operative driver. In vivo, dual mTOR inhibition (PP242) reduces EZH2, reduces H3K27me3, and increases p21 in GBM xenografts, with GSKJ4 co-treatment partially reversing PP242’s antitumor effect. This dual-input architecture, mTORC1 regulating the enzyme, mTORC2 independently regulating its substrate, may explain why EZH2 inhibition or mTOR inhibition alone is often incomplete, and supports combinatorial targeting of EZH2 alongside mTOR or methionine/SAM metabolism in *EGFR*-mutant GBM [89].

**Figure 4:**
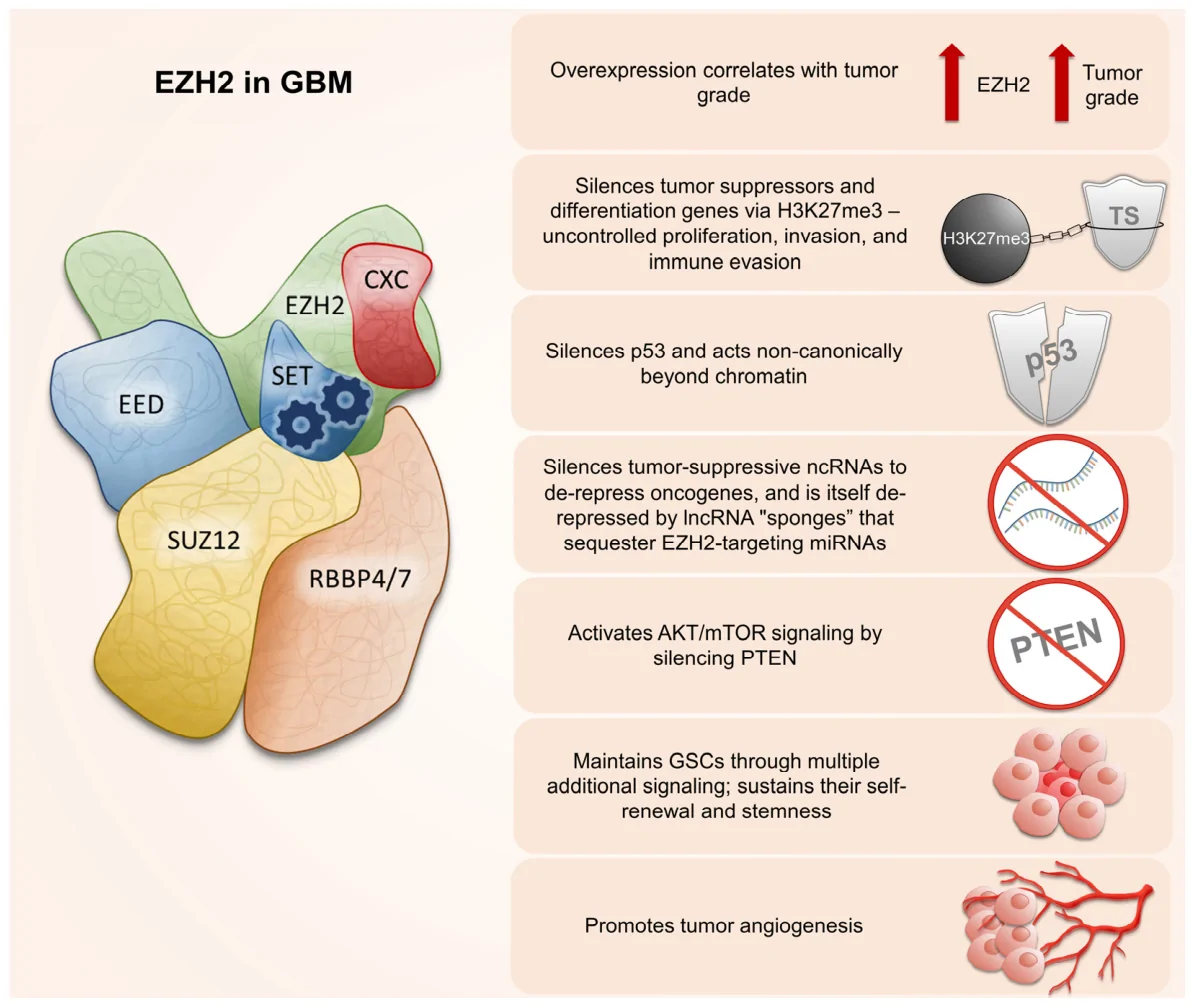
EZH2 in glioblastoma (GBM). The left panel illustrates the core composition of the Polycomb Repressive Complex 2 (PRC2), consisting of EZH2, EED, SUZ12, RBBP4/7, and a catalytic SET domain-containing EZH2 subunit. The right panel summarizes the major mechanisms through which EZH2 contributes to GBM progression. TS—tumor suppressors, GSCs—glioma stem cells.

Taken together, these findings position EZH2 as a convergent oncogenic hub in GBM. Its activity is driven by a diverse repertoire of upstream regulators, including signaling scaffolds, kinases, and ncRNA-mediated circuits. These mechanisms contribute to multiple oncogenic programs, including PTEN/AKT-Wnt/β-catenin activation, glycolytic reprogramming, stem-cell self-renewal, immune modulation, cytoskeletal remodeling, and ncRNA-driven feedback loops. Importantly, EZH2 abundance and catalytic activity can be regulated independently, through mTORC1 and mTORC2/SAM signaling, respectively. In addition, some oncogenic functions of EZH2 are methylation-independent, such as Twist1 mRNA stabilization. Therefore, catalytic EZH2 inhibitors alone may not fully suppress its tumor-promoting effects. These observations support combinatorial therapeutic strategies that couple EZH2 inhibition with targeting of upstream regulators (e.g., mTOR, MELK) or downstream effectors (e.g., HIF1α, CXCR4, Twist1), rather than relying on monotherapy. The summary of EZH2 properties is presented in Figure 4.

## 4. Clinical Relevance of EZH2 in GBM and Other Gliomas

Several studies have investigated the clinical significance of EZH2 in glioma using patient-derived tissue samples and publicly available transcriptomic datasets. Although most studies focused on gliomas as a heterogeneous group rather than GBM specifically, their findings consistently indicate that EZH2 expression increases with tumor grade and may have prognostic relevance.

Zhang et al. performed a meta-analysis of six independent studies comprising 575 glioma patients to evaluate the prognostic significance of EZH2 expression [90]. The analysis included studies involving WHO grade I-IV gliomas and assessed EZH2 expression primarily by immunohistochemistry. The authors found that elevated EZH2 expression was associated with significantly poorer overall survival (HR = 2.23) and progression-free survival. Additionally, high EZH2 expression correlated with lower Karnofsky Performance Status scores. Since the meta-analysis pooled data from gliomas of different grades, the findings support a prognostic role of EZH2 in glioma overall, although they do not specifically establish its prognostic value in GBM alone.

Chen et al. conducted a comprehensive analysis using several large glioma cohorts, including data from TCGA and CGGA, complemented by validation in additional clinical specimens [91]. The study demonstrated that EZH2 expression increased progressively with tumor grade and was highest in high-grade gliomas, including GBM. Survival analyses showed that patients with elevated EZH2 expression had significantly shorter survival than those with lower expression levels. The authors proposed EZH2 as an independent prognostic biomarker for glioma. Importantly, while GBM was included in the analyses, the reported prognostic associations were largely derived from combined glioma cohorts rather than GBM-specific cohorts.

Wong et al. investigated EZH2 protein expression in a cohort of human glioma specimens using immunohistochemistry [92]. The study assessed the relationship between EZH2 protein levels, clinicopathological features, IDH1 status, and patient survival. High EZH2 expression was associated with higher tumor grade and adverse prognosis, particularly in IDH1 R132H-negative gliomas. The authors identified EZH2 as a marker of poorer clinical outcome and suggested its potential utility in prognostic stratification. Similar to the studies above, the conclusions primarily refer to gliomas broadly, with GBM representing a subset of the analyzed tumors rather than a separate study population.

More recently, Peng et al. combined analyses of transcriptomic data from public databases with immunohistochemical evaluation of 147 clinical glioma samples [93]. The investigators reported significantly elevated EZH2 expression in tumor tissue compared with normal brain tissue and observed increasing expression with advancing tumor grade. In survival analyses, high EZH2 expression was associated with poorer overall survival, disease-specific survival, and progression-free interval, particularly in high-grade gliomas. The study concluded that EZH2 may serve as a clinically relevant prognostic biomarker in glioma. Although GBM samples were included among the high-grade tumors, the prognostic analyses were performed primarily at the level of glioma and high-grade glioma cohorts, rather than exclusively in GBM patients.

**Figure 5:**
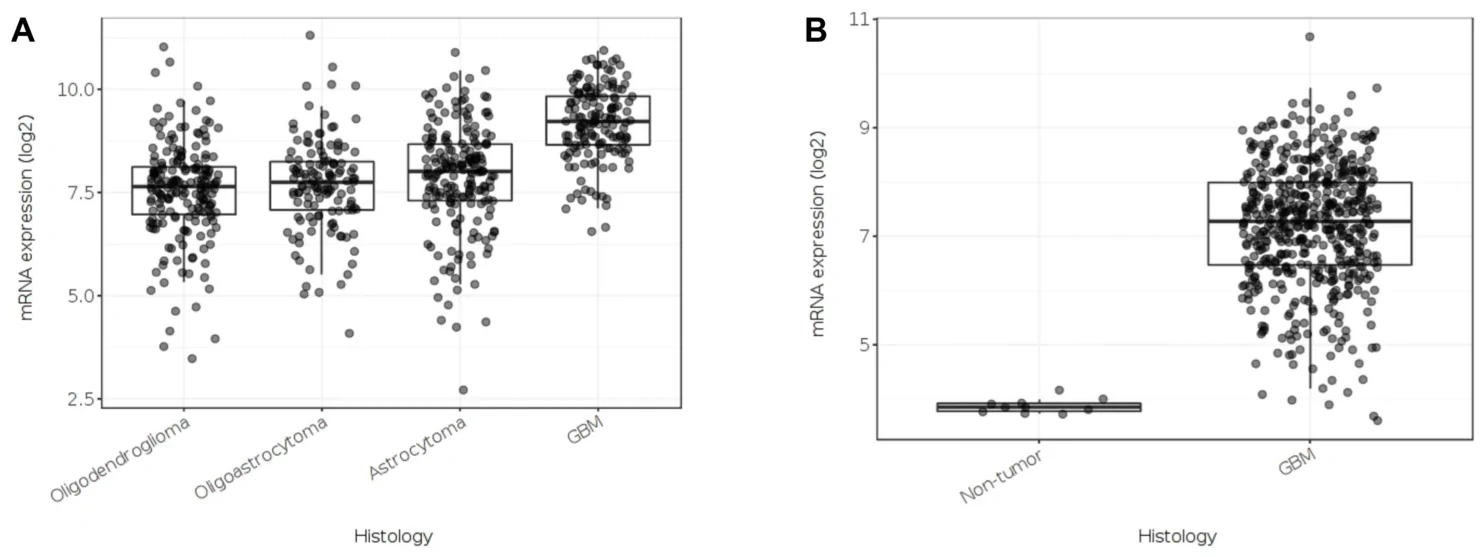
*EZH2* mRNA expression across glioma histological subtypes and in glioblastoma (GBM) versus non-tumor brain tissue. (**A**) Distribution of *EZH2* mRNA expression (log2-transformed) in oligodendroglioma, oligoastrocytoma, astrocytoma, and GBM samples. (**B**) Comparison of *EZH2* mRNA expression (log2-transformed) between non-tumor brain tissue and GBM samples. Data are presented as box-and-whisker plots with individual samples overlaid as points. The central line represents the median, boxes indicate the interquartile range (IQR), and whiskers extend to 1.5 × IQR.

To further characterize EZH2 expression across glioma subtypes, publicly available transcriptomic data were analyzed and visualized according to histological classification. As shown in Figure 5A, EZH2 mRNA was detected in all examined glioma subtypes, including oligodendroglioma, oligoastrocytoma, astrocytoma, and GBM. The distribution of expression values varied among tumor types, with GBM samples exhibiting the highest median expression levels compared with the lower-grade glioma subgroups.

Comparison of non-tumor brain tissue and GBM samples (Figure 5B) demonstrated substantially higher EZH2 mRNA expression in GBM. While expression levels in non-tumor tissues were consistently low and showed limited variability, GBM samples displayed markedly elevated expression together with a broader range of values. These data indicate increased EZH2 transcript abundance in GBM relative to non-neoplastic brain tissue and suggest differences in EZH2 expression across glioma histological subtypes.

**Figure 6:**
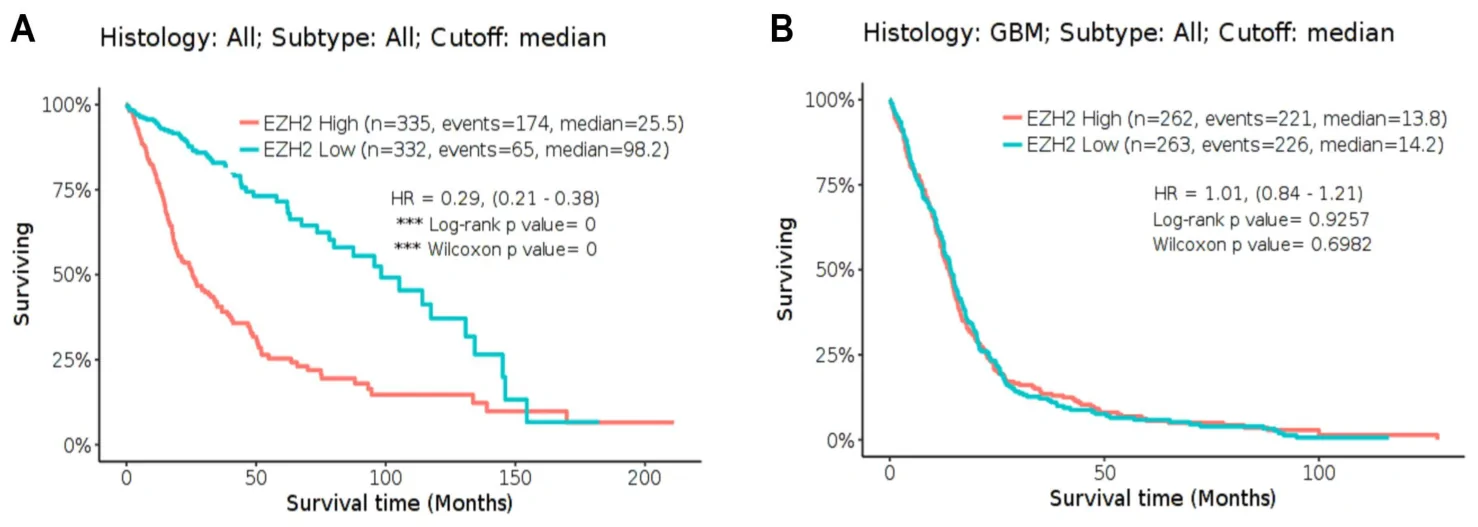
Kaplan–Meier overall survival curves stratified according to *EZH2* mRNA expression in the (**A**) TCGA-GBMLGG and (**B**) TCGA-GBM cohorts. In each dataset, patients were divided into high- and low-expression groups using the median *EZH2* expression value as the cutoff. Survival analyses were performed using the GlioVis platform. The corresponding hazard ratios (HRs), median survival times, and log-rank test results are shown in the figure. *** indicates a statistically significant difference between the compared groups.

To further assess the clinical relevance of EZH2 expression in glioma, particularly GBM, we analyzed publicly available survival data from the TCGA-GBMLGG and TCGA-GBM cohorts using the GlioVis platform. Kaplan–Meier survival analysis of the combined TCGA-GBMLGG cohort demonstrated a significant association between elevated EZH2 mRNA expression and reduced overall survival. Patients with high EZH2 expression exhibited a markedly shorter median survival time compared with those with low expression (25.5 vs. 98.2 months, respectively). In contrast, analysis of the TCGA-GBM cohort alone revealed no significant relationship between EZH2 expression and patient outcome. The high- and low-expression groups displayed nearly identical survival curves, with comparable median survival times (13.8 vs. 14.2 months, respectively; HR = 1.01, 95% CI: 0.84–1.21; log-rank *p* = 0.9257) (Figure 6B). These findings suggest that the prognostic value of EZH2 expression is evident in the broader glioma population represented by the GBMLGG cohort, but is not retained in patients with GBM alone, indicating that the association between EZH2 expression and survival may depend on glioma grade and molecular composition.

## 5. EZH2 and KDM1A Crosstalk

EZH2 can be recruited to lncRNAs alongside lysine-specific demethylase 1 (KDM1A/LSD1) to regulate gene expression. KDM1A is an FAD-dependent amine oxidase and the first histone demethylase identified, establishing that histone methylation is a dynamic, reversible mark rather than a permanent one. Its enzymatic activity is restricted to mono- and di-methylated lysines and is entirely context-dependent: within CoREST, KDM1A demethylates H3K4me1/2 to silence transcription, whereas in complexes with nuclear hormone receptors or lineage-specific transcription factors it instead targets the repressive H3K9me1/2 mark, thereby activating transcription [94,95]. This bidirectionality, together with non-histone substrates such as p53, DNMT1, and E2F1, positions KDM1A as a broad, context-tunable regulator of gene expression rather than a strict repressor. In GBM, KDM1A expression rises with tumor grade, sustains GSC self-renewal and stemness programs (via SOX2, OLIG2, and MYC-driven transcriptional output), supports metabolic flexibility through lipogenic and integrated-stress-response pathways, and is stabilized by upstream regulators including the GSK3β–USP22 and USP7 axes [96,97,98,99,100,101,102]. Much of this oncogenic activity is channeled through partnerships with lncRNA scaffolds, most notably HOTAIR, that recruit KDM1A to specific genomic loci, though not all HOTAIR-driven phenotypes require KDM1A [103,104].

Although KDM1A and EZH2 catalyze opposite biochemical reactions, accumulating evidence demonstrates that these enzymes frequently cooperate to establish and maintain transcriptionally repressive chromatin. Rather than acting independently, both proteins are often recruited to the same genomic loci, where their complementary enzymatic activities reinforce stable gene silencing [105]. KDM1A removes activating H3K4me1/2 marks, whereas EZH2 deposits the repressive H3K27me3 modification. The simultaneous elimination of activating histone marks and the accumulation of repressive modifications create a chromatin environment that is highly resistant to transcriptional activation [106,107,108,109]. Functional cooperation between KDM1A and EZH2 has been described during embryonic development [106,110,111,112] and in multiple cancers, where co-occupancy of KDM1A- and PRC2-containing complexes at promoters suppresses neural differentiation programs while sustaining proliferative and stemness-related transcriptional networks [55,99]. Accordingly, growing experimental evidence suggests that simultaneous inhibition of KDM1A and EZH2 often yields stronger antitumor effects than inhibition of either enzyme alone [113,114].

However, in GBM, EZH2 and KDM1A are not always obligate joint actors. While they are frequently recruited to the same lncRNA scaffolds, the resulting relationship is locus- and context-dependent: depending on the target gene and phenotype, the two enzymes can act jointly, or the scaffold can selectively engage just one of them while leaving the other dispensable. Importantly, this cooperation is not mediated by a direct physical interaction between KDM1A and EZH2 themselves; rather, both enzymes are independently recruited to a shared lncRNA scaffold, which brings their activities into spatial proximity without requiring a direct protein–protein contact between them. HOTAIR is the clearest illustration of this flexibility. As a scaffold, it is capable of binding both PRC2/EZH2 and the KDM1A-REST-CoREST complex physically, in principle allowing a single RNA molecule to organize a multi-enzyme repression platform combining H3K27 trimethylation with H3K4 demethylation [104,115,116]. In practice, however, functional studies show that HOTAIR engages these two partners selectively rather than jointly, depending on the downstream phenotype. In GBM cell-cycle control, EZH2 is the operative effector and KDM1A is dispensable: the indirect EZH2 inhibitor DZNep reduces HOTAIR expression and induces G1 arrest, whereas KDM1A knockdown or inhibition does not alter HOTAIR levels or cell-cycle progression. Importantly, DZNep acts indirectly by perturbing cellular S-adenosylmethionine metabolism and can affect multiple methyltransferases. Therefore, effects observed with DZNep cannot be attributed exclusively to EZH2 inhibition and should be interpreted as pharmacological evidence consistent with EZH2 involvement rather than as definitive evidence of EZH2-specific inhibition [117,118]. Nevertheless, the functional distinction is supported by the observation that DZNep, but not the KDM1A inhibitor 2-PCPA, mimicks HOTAIR knockdown, induces p16/p21 upregulation, cyclin D1/E downregulation, and G1/S arrest. Only HOTAIR’s PRC2-binding domain (not its KDM1A-binding domain) rescues this arrest, and only EZH2 inhibition suppresses xenograft growth in vivo [118]. The reverse pattern is observed in epithelial cell migration. HOTAIR’s ability to promote a migratory, mesenchymal-like state depends specifically on its KDM1A-binding domain, not its PRC2-binding domain, with HOTAIR altering KDM1A occupancy at promoters and shifting H3K27ac while leaving H3K4me2 largely unchanged [103]. While KDM1A itself is a demethylase, its presence in the KDM1A-CoREST-HDAC complex makes H3K27ac dynamic shifts a primary hallmark of its activity at active gene promoters and enhancers. Collectively, HOTAIR does not function as an obligate dual-enzyme complex, rather, it partitions distinct oncogenic outputs to distinct partners, recruiting EZH2 for cell-cycle control and KDM1A for migratory/mesenchymal phenotypes, with each enzyme sufficient on its own for its respective function.

The AGAP2-AS1 scaffold presents a different model—one of genuine joint dependence. Here, the lncRNA physically scaffolds both KDM1A and EZH2 simultaneously at the *TFPI2* promoter. EZH2 deposits H3K27me3, and KDM1A removes H3K4me2, creating a combined repressive chromatin state that silences this tumor suppressor. Knockdown of AGAP2-AS1 or inhibition of either KDM1A or EZH2 individually restores TFPI2 expression and reduces proliferation and invasion while increasing apoptosis [119]. Because disrupting either enzyme alone is sufficient to relieve repression at this locus, the two enzymes’ contributions are additive rather than redundant. Unlike the HOTAIR examples above, each is independently necessary for the full repressive effect.

Ultimately, KDM1A–EZH2 crosstalk in GBM is not a single, fixed relationship but a locus- and context-dependent one, mediated by shared lncRNA scaffolds rather than by direct physical interaction between the enzymes themselves. At the AGAP2-AS1/TFPI2 axis, both enzymes are jointly required. At the HOTAIR axis, the same scaffold instead partitions cell-cycle control to EZH2 and migratory control to KDM1A, with each acting independently of the other. This distinction has direct therapeutic relevance: combination KDM1A/EZH2 inhibition is most clearly justified where dual-enzyme dependence has been demonstrated, such as AGAP2-AS1-driven TFPI2 silencing, whereas HOTAIR-driven phenotypes may instead call for enzyme-specific targeting matched to the relevant downstream function (EZH2 inhibition for cell-cycle effects, KDM1A inhibition for invasive/mesenchymal effects).

**Figure 7:**
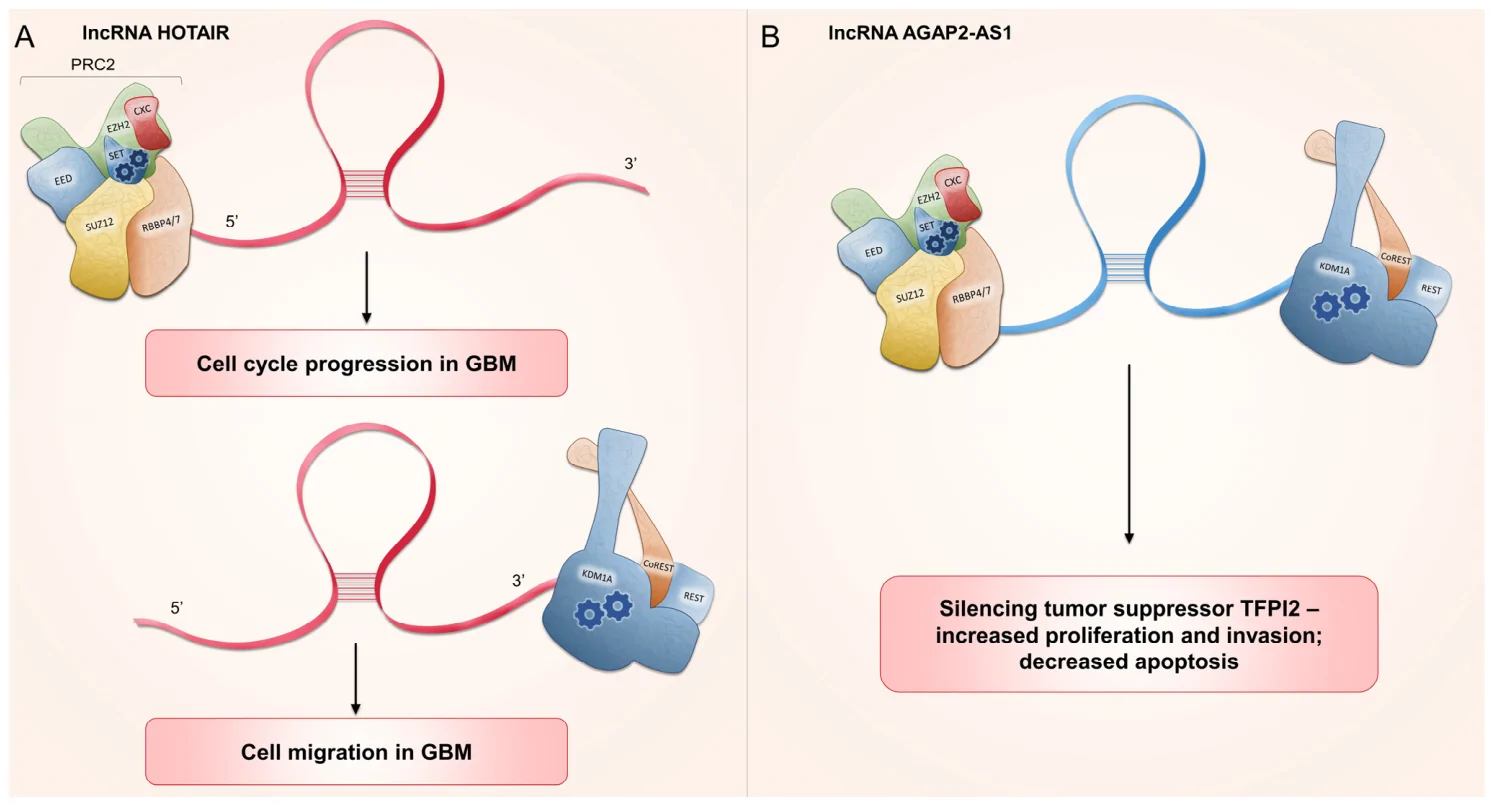
(**A**) Interaction of long non-coding RNA (lncRNA) HOTAIR with EZH2 (in PRC2) and KDM1A (with CoREST and REST) respectively. (**B**) Interaction of lncRNA AGAP2-AS1 with both EZH2 (in PRC2) and KDM1A (with CoREST and REST). PRC2—Polycomb Repressive Complex 2, GBM—glioblastoma.

Nevertheless, available evidence supporting dual EZH2/KDM1A inhibition in GBM remains limited and derives predominantly from preclinical studies performed in selected experimental models. To date, no study has systematically compared responses across the established molecular GBM subtypes and whether therapeutic synergy is subtype-specific remains unknown. Likewise, convincing evidence for true pharmacological antagonism has not been reported, although context-dependent differences in sensitivity suggest that combined inhibition is unlikely to be universally beneficial. Future studies incorporating molecular stratification will therefore be essential to define the patient populations most likely to benefit from dual epigenetic targeting [86,120,121]. Table 2 below summarizes HOTAIR and AGAP2-AS1 regulatory networks governing KDM1A and EZH2 in GBM. Furthermore, visual representation of interactions of EZH2 with HOTAIR and AGAP2-AS1 is depicted in Figure 7 below.

## 6. Therapeutic Targeting of EZH2

### 6.1. First-Generation Selective EZH2 Catalytic Inhibitors

The most extensively studied class of EZH2 inhibitors comprises SAM-competitive compounds that target the catalytic SET domain, thereby suppressing PRC2 activity by inhibiting H3K27 trimethylation [122]. The development of this class began with EPZ005687, one of the first highly selective EZH2 inhibitors, which provided proof of concept that pharmacological inhibition effectively reduces H3K27me3 levels and suppresses tumor cell growth [123,124]. Although its clinical advancement was limited by suboptimal pharmacokinetic properties, EPZ005687 paved the way for second-generation inhibitors, including GSK343, GSK126, and tazemetostat [123,125,126].

Within this class, GSK343 demonstrated substantial antitumor activity in glioma models. Yu et al. reported that GSK343 significantly inhibited the proliferation of GBM cell lines and induced G0/G1 cell-cycle arrest, alongside reducing migratory and invasive phenotypes and downregulating mesenchymal marks associated with aggressive disease. GSK343 also impaired neurosphere formation and reduced the self-renewal capacity of GSCs, indicating that EZH2 inhibition may directly target the stem cell compartment responsible for tumor maintenance [127]. Expanding on this mechanistic landscape, Scuderi et al. investigated GSK343 with a focus on canonical and non-canonical NF-κB/IκBα signaling and innate immune modulation. In vitro, GSK343 reduced the viability of three GBM cell lines in a concentration-dependent manner while sparing normal astrocytes. Mechanistically, it suppressed both arms of NF-κB signaling by reducing NF-κB, IKKβ, and NIK while restoring IκBα, downregulating IL-1β and TNF-α, and reversing EMT markers. Furthermore, GSK343 shifted the apoptotic balance toward Bax/p53 over Bcl2. These findings were confirmed in a U87 xenograft model, where GSK343 reduced oxidative stress markers and restored superoxide dismutase (SOD) levels. Notably, ex vivo studies in patient-derived GBM cells revealed upregulation of the natural killer (NK) cell-recruiting chemokines CXCL9, CXCL10, and CXCL11, suggesting that EZH2 inhibition may partially restore innate antitumor immune surveillance. A key unresolved limitation, however, is that GSK343’s BBB penetration has not been directly demonstrated in vivo [128].

GSK126 is another direct EZH2 inhibitor that has been evaluated in preclinical GBM models. Grinshtein et al. [48] showed that GSK126 reduced the viability of a subset of patient-derived brain tumor-initiating cell (BTIC) models, although these effects varied across BTIC models. Its translational application as a monotherapy is frequently limited by unfavorable pharmacokinetic properties and restricted BBB penetration due to active efflux transporters such as ABCB1 and ABCG2 [21,129]. However, Zhao et al. demonstrated that GSK126 occupies the SAM-binding site in the SET domain of EZH2, thereby disrupting EZH2-driven stemness in GSC models, while genetic silencing of EZH2 impaired stemness. GSK126 dose-dependently recapitulated these effects, suppressing H3K27me3 and downregulating Notch1, NICD, HES1, CD133, and NESTIN. This led to reduced self-renewal capacity in limiting dilution assays and decreased invasion in transwell assays. Critically, these in vitro findings were supported by in vivo efficacy: GSK126 reduced subcutaneous xenograft tumor weight and, in an orthotopic intracranial model, significantly prolonged mouse survival without causing major changes in body weight, indicating its antitumor activity in this model [69].

Tazemetostat (EPZ-6438) was the most clinically advanced EZH2 inhibitor and the first to receive FDA approval—in epithelioid sarcoma (January 2020) and relapsed/refractory follicular lymphoma (June 2020). In a first-in-human phase I trial (NCT01897571), tazemetostat demonstrated an acceptable safety profile and antitumor activity, supporting further evaluation in hematologic malignancies and solid tumors. Subsequent studies (e.g., NCT02601950, NCT02860286) confirmed activity in epithelioid sarcoma and other indications, including mesothelioma [130]. However, in March 2026 the sponsor voluntarily withdrew tazemetostat from all indications and markets following an Independent Data Monitoring Committee recommendation in the confirmatory SYMPHONY-1 trial (NCT04224493), which showed an excess of secondary hematologic malignancies (mainly MDS/AML); all active trials and expanded access programs were halted and the FDA formally withdrew approval of the NDA effective 22 June 2026, at the sponsor’s request [131,132]. Despite these advances in non-CNS tumors, tazemetostat’s efficacy as a single agent in GBM remains limited, and no further clinical development of this compound in glioma is currently ongoing.

### 6.2. Dual EZH1/EZH2 Inhibitors

A major limitation of selective EZH2 inhibition is functional redundancy with its homolog EZH1, which can partially compensate for EZH2 loss. This observation has led to the development of dual EZH1/EZH2 inhibitors to achieve more complete suppression of PRC2 activity [133,134]. Within this approach, UNC1999 stands as the most extensively studied dual inhibitor in GBM. In a large-scale epigenetic drug screen, Grinshtein et al. identified UNC1999 as one of the most potent compounds against patient-derived BTICs. The compound exhibited low-micromolar cytotoxicity across genetically heterogeneous GBM models, significantly reduced self-renewal capacity, and effectively decreased H3K27me3 levels. Importantly, UNC1999 induced cell-cycle arrest and demonstrated enhanced antitumor activity when combined with dexamethasone, resulting in reduced tumor growth in vivo. These observations suggest that simultaneous inhibition of EZH1 and EZH2 may overcome adaptive resistance mechanisms associated with selective EZH2 blockade [48].

Valemetostat (DS-3201) is another clinically developed dual EZH1/EZH2 inhibitor that exerts potent, sustained inhibition of PRC2 activity. Although direct evidence in GBM remains limited, early-phase clinical studies (NCT02732275, NCT04703192) have demonstrated encouraging activity in hematological malignancies, supporting its favorable pharmacodynamic profile and clinical feasibility [123]. Given its unique ability to circumvent functional compensation by EZH1, valemetostat represents a promising candidate for future translational investigation in GBM and other solid tumors.

### 6.3. Targeted EZH2 Degraders

Because conventional EZH2 inhibitors only block enzymatic activity, they often fail against cancers in which EZH2 acts via non-catalytic pathways independent of its methyltransferase function. To address this, investigators have designed targeted degraders to eliminate the physical protein structure entirely. A landmark study by Ma et al. [135] described MS1943, the first selective EZH2 degrader. Unlike conventional inhibitors, MS1943 induced substantial depletion of cellular EZH2 protein levels, resulting in pronounced antitumor activity in vitro and in vivo. MS1943 effectively reduced EZH2 expression, suppressed H3K27me3 levels, inhibited tumor cell proliferation, and induced apoptosis across multiple models. Notably, the antitumor effects of EZH2 degradation were more pronounced than those achieved by catalytic inhibition alone, suggesting that complete elimination of EZH2 may overcome the limitations associated with catalytically active EZH2 inhibitors.

Although EZH2 degraders have not yet been widely deployed in neuro-oncology, a second key molecule has recently been validated in glioma. Covalent EZH2-targeting compounds can overcome the limitations of standard agents that fail against non-methyltransferase functions, such as EZH2’s transcriptionally driven activation of CDK4 independent of PRC2 in triple-negative breast cancer, or its role in EZH2-mutant lymphoma [136]. Using this degrader rationale, Zhang et al. evaluated IHMT-337, a covalent EZH2-targeting agent that irreversibly binds to Cys663 and promotes EZH2 degradation via the CHIP-ubiquitin pathway. In vitro, IHMT-337 showed potent activity against glioma cells, with EZH2 protein levels declining dose-dependently alongside restored SLC12A5 expression and suppressed WNK1 phosphorylation. Functionally, IHMT-337 inhibited glioma cell migration (including synergistic effects when combined with WNK1 or NKCC1 inhibitors), suppressed proliferation, and arrested cell-cycle progression, though it did not significantly induce apoptosis. Additionally, BBB permeability of IHMT-337 was demonstrated in vitro using an hCMEC/D3 Transwell model. In an orthotopic glioma xenograft model, it successfully reduced tumor growth, lowered EZH2 and Ki-67 expression while restoring KCC2, and significantly extended survival compared to controls, confirming the therapeutic potential of targeting the SLC12A5-KCC2 axis via EZH2 destruction [71].

### 6.4. Disruptors of EZH2 Interactions

An alternative to degrading the enzyme is disrupting its physical interactions with oncogenic lncRNAs or co-factors, which frequently drive therapeutic resistance. A novel class of small-molecule inhibitors targeting the HOTAIR/EZH2 and PRADX/EZH2 structural complexes emerged as a powerful strategy to reverse TMZ resistance in GBM across three distinct candidate molecules.

Designed to disrupt the HOTAIR/EZH2 interaction, EPIC-0628 increases ATF3 expression, which in turn suppresses *MGMT* transcription by blocking the recruitment of p300, p-p65, p-Stat3, and SP1 to the *MGMT* promoter. It simultaneously induces CDKN1A/p21 to arrest cells in G1 and inhibits homologous recombination (HR) repair through the ATF3-p38-E2F1 axis. In GBM cell lines and patient-derived cells, EPIC-0628 reduced proliferation and enhanced DNA damage. When it was combined with TMZ, it significantly increased γH2AX accumulation, apoptosis, and growth suppression. These findings were confirmed in vivo, where the combination reduced intracranial and subcutaneous tumor growth, lowered Ki-67 expression, and prolonged survival without systemic toxicity [137].

EPIC-0412 similarly disrupts the HOTAIR/EZH2 interaction but reprograms resistance via twin pathways. It upregulates *CDKN1A* and *BBC3* to drive p21-mediated G1 arrest and PUMA-associated apoptosis, while suppressing the p21-E2F1 DNA damage repair axis and downregulating core HR repair genes (*RAD50, RAD51, MRE11, CHEK1*, and *CHEK2*). Additionally, it lowers MGMT expression in MGMT-positive tumors by activating ATF3, increasing UBXN1, inhibiting p-p65, and recruiting HDAC1 to the MGMT promoter, which subsequently reduces H3K27ac and weakens MGMT-mediated repair. In both MGMT-negative and MGMT-positive GBM models, EPIC-0412 enhanced TMZ-induced DNA damage, increased γ-H2AX, and prolonged survival in tumor-bearing mice [138].

EPIC-0307 targets the alternative PRADX/EZH2 interaction. In GBM cells, it selectively disrupts PRADX binding to EZH2, increasing p21 and PUMA levels to trigger G1 arrest and apoptosis, while weakening DNA repair by downregulating the HR genes *RAD50, RAD51, CHK1, CHK2,* and *MRE11*. It further enhances TMZ efficacy by epigenetically suppressing MGMT in MGMT-positive tumors via the ATF3-pSTAT3-HDAC1 pathway, reducing MGMT transcription and increasing TMZ-induced DNA lesions. In vitro and in vivo models demonstrated reduced tumor growth, increased γH2AX, and extended survival, with the most robust benefits observed when combined with TMZ [139].

Targeting a similar lncRNA axis, Shi et al. demonstrated that the HOTAIR-EZH2 interaction inhibitor AC1Q3QWB (AQB) suppresses glioma growth by reactivating the tumor suppressor CWF19L1 through H3K27ac-dependent chromatin changes. In glioma cell lines and patient-derived xenograft (PDX) models, AQB increased CWF19L1 expression, reduced CDK4/CDK6 and p-RB signaling, and caused G1 arrest, indicating that CWF19L1 functions as a crucial cell-cycle brake. The authors discovered that AQB and the CDK4/6 inhibitor palbociclib act synergistically, producing significantly stronger inhibition of proliferation, migration, invasion, and Wnt/β-catenin signaling than either agent alone. In vivo, this combination reduced intracranial tumor burden, lowered Ki67, CDK4, and CDK6 expression, and improved survival in GBM PDX mice, establishing a strong rationale for dual targeting in tumors characterized by high HOTAIR/EZH2 and low CWF19L1 expression [140].

### 6.5. Combinatorial Strategies

Given the dense network of compensatory signaling pathways in GBM, monotherapy with EZH2 inhibitors often yields limited clinical efficacy, prompting the exploration of multi-target combination regimens.

Freitag et al. identified the combination of EZH2 inhibition and CDK4/6 inhibition as a highly promising therapeutic strategy. Utilizing the selective EZH2 inhibitor GSK126 alongside the CDK4/6 inhibitor abemaciclib across patient-derived 2D cultures, 3D spheroids, organoids, and in ovo systems, the combination exhibited potent synergistic antitumor activity. This dual blockade significantly reduced cell viability and invasion, reversed stem-like and mesenchymal features, and disrupted endoplasmic reticulum-mitochondrial homeostasis. Mechanistically, this led to severe mitochondrial dysfunction, elevated reactive oxygen species (ROS), activation of the unfolded protein response (UPR), and impaired mitochondrial respiration. Transcriptional and gene-expression analyses showed profound downregulation of programs involved in cell-cycle progression, mitosis, DNA repair, invasion, and stemness, alongside shifts toward a more differentiated state and suppression of pathways linked to Wnt/β-catenin signaling and immunosuppression. The authors noted that the clinically approved EZH2 inhibitor tazemetostat also synergized with abemaciclib, though less robustly than GSK126 [141].

Mishra et al. proposed a combination strategy pairing the PI3K inhibitor PI-103 with the EZH2 inhibitor EPZ-6438 to suppress tumor aggressiveness through complementary pathways. In U-87 GBM cells, dual treatment reduced migration and invasion, altered actin and tubulin organization, weakened cell adhesion, lowered ROS, and blocked the G1-to-S transition without inducing overt apoptosis at tested doses. It diminished MMP2 activity, CD24 expression, and the EMT-associated markers β-catenin, SNAIL3, and SMAD1, while reducing sphere formation and colony growth. Additionally, the combination altered the TME by reducing angiogenic signaling in conditioned media, impairing endothelial tube formation, and modulating metastasis- and inflammation-associated cytokines, including VCAM-1, ST2, CD30, lipocalin-2, Ang2, IL-10, and IL-24 [142].

Zhu et al. demonstrated that PTEN deficiency in GBM suppresses type I interferon signaling and creates an immunosuppressive TME that weakens antitumor T-cell activity. PTEN loss reduces endogenous retrovirus (ERV)-derived dsRNA and MAVS-dependent interferon responses, while increasing EZH2 activity and H3K27me3, which helps silence ERVs and limits the viral mimicry response needed for immune activation. While the DNMT inhibitor 5-azacytidine alone could induce this response in PTEN-intact tumors, it was much less effective in PTEN-deficient models. However, combining 5-azacytidine with EZH2 inhibition (via tazemetostat) restored ERV expression, boosted interferon-stimulated genes, and increased dsRNA accumulation. In mouse GBM models, this dual epigenetic therapy successfully reprogrammed the TME toward an inflamed, T-cell-rich state, reduced regulatory T cells (Tregs), and significantly improved tumor control and survival [50].

Sharma et al. designed a first-in-class dual EZH2–HSP90 inhibitor for TMZ-resistant GBM [143]. HSP90 is a molecular chaperone responsible for stabilizing numerous oncogenic client proteins driving GBM, including EGFR, AKT, CDK4, and MET. Its inhibition induces radiosensitization, blocks DNA repair, and disrupts redox homeostasis [144,145,146]. Appending an HSP90-inhibitory resorcinol pharmacophore to the tazemetostat scaffold yielded compound 7. Critically, anti-GBM activity was observed only in compounds that simultaneously inhibited both targets. Mechanistically, compound 7 suppressed kinetochore/spindle gene expression, downregulated centromere proteins (CENP), induced G2/M arrest, impaired DNA repair, and depleted ROS-scavenging proteins, leading to an accumulation of mitochondria-derived ROS and caspase-3 activation. In vivo, compound 7 effectively suppressed TMZ-resistant xenograft growth, whereas tazemetostat alone had no effect. Crucially, the single dual-targeting scaffold bypassed the lethal systemic toxicity caused by the standard two-drug combination doublet [143].

Zhang et al. explored a combination strategy based on the observation that H3K27M-mutant gliomas exhibit clinical responsiveness to the imipridone compound ONC201, an activator of the ISR and TRAIL-DR5 apoptotic pathway. The authors hypothesized that pharmacological reduction of H3K27 methylation via EZH2 inhibition could mimic this mutation and sensitize GBM cells to ONC201. In the U251 GBM cell line, both tested EZH2 inhibitors, tazemetostat and PF-06821497, synergized with ONC201 to suppress cell viability, yielding combination index values below 0.3 at specific dosages. Mechanistically, the combination enhanced the induction of ATF4 and downstream DR5 relative to either agent alone, and ATF4 knockdown rescued cells from apoptosis. Adding the HDAC inhibitor vorinostat to the EZH2i-ONC201 combination further enhanced apoptosis, consistent with cooperative chromatin opening through simultaneous H3K27 demethylation and acetylation. Across all conditions, synergistic cell death correlated directly with reduced H3K27me3 rather than increased H3K27ac, suggesting that EZH2-mediated repression of ISR-responsive loci is the primary sensitizing mechanism [147].

Alexanian and Brannon demonstrated that DZNep, an indirect EZH2 inhibitor, inhibits S-adenosylhomocysteine hydrolase, thereby depleting the methyl donor S-adenosylmethionine and reducing EZH2 protein levels and global H3K27me3 without directly blocking EZH2’s active site. DZNep showed synergistic interactions with the G9a methyltransferase inhibitor BIX01294 and the HDAC inhibitor trichostatin A (TSA), supporting the concept that simultaneous targeting of multiple epigenetic pathways enhances antitumor activity. In U87 GBM cells, these agents cooperatively suppressed viability at concentrations that individually produced only moderate effects. Notably, the TSA + BIX01294 combination at intermediate concentrations yielded the greatest reduction in U87 viability (to 2–3% of control) while exerting minimal effects on normal adipose-derived mesenchymal stem cells. DZNep alone showed limited selectivity, but its inclusion in the overall epigenetic combination strategy further supported synergistic GBM cell killing and highlighted the potential benefit of concurrent inhibition of EZH2-, G9a-, and HDAC-dependent pathways [148].

De la Rosa et al. targeted EZH2 with DZNep alone and in combination with the HDAC inhibitor panobinostat and TMZ across GBM lines, TMZ-resistant sublines, and primary cultures. DZNep plus panobinostat was the most synergistic pairwise combination, reducing colony formation and increasing apoptosis (BAX/NOXA upregulation, caspase 3/7 activation) in most lines, consistent with the rationale that EZH2 inhibition relieves H3K27me3-mediated repression, sensitizing chromatin to panobinostat’s HDAC blockade. DZNep combined with TMZ showed weaker synergy, likely reflecting slower EZH2 inhibition kinetics relative to TMZ’s rapid DNA-damaging action. However, the triple combination (DZNep + panobinostat + TMZ) was strongly synergistic overall, with effects most pronounced in p53/PTEN-mutant lines [149]. The second study situated this response within the dynamics of p53 signaling. EZH2 inhibition stabilizes p53 by blocking its ubiquitin-mediated degradation and activates downstream effectors such as MDM2, p21, and GADD45 in wild-type p53 cells. Consistent with this, DZNep increased p53 expression by Western blot in wild-type lines but not in mutant p53 lines, which are typically more DZNep-resistant. When combined with the p53-reactivating agent APR-246, DZNep produced only an additive effect, which the authors attribute to convergent p53 stabilization in wild-type lines and to p53-independent oxidative stress. This lack of synergy stemmed from APR-246’s failure to reactivate mutant p53 in this specific system, underscoring that EZH2 inhibition is a mechanistically versatile axis whose efficacy is strongest when paired with complementary chromatin-opening pathways, such as HDAC inhibition [150].

### 6.6. Non-Canonical Activity and microRNA Regulation

Beyond standard nuclear repression, recent breakthroughs have highlighted non-canonical functions of EZH2 and endogenous microRNA networks that dictate GBM stemness and cell death. A recent study by Doualle et al. used a dedifferentiated GBM stem cells (dGSCs) model to study therapeutic resistance and found that EZH2 inhibition can overcome this resistance specifically via ferroptosis. dGSCs showed reduced sensitivity to conventional cytotoxic drugs and to ferroptosis induction (via RSL3), linked to decreased proliferation, increased STAT3/Akt activation, and enhanced oxidative stress protection. This resistant phenotype was directly associated with reduced nuclear EZH2 but increased EZH2 phosphorylation at serine 21, which effectively redirects EZH2 toward non-canonical, H3K27me3-independent functions. The EZH2 inhibitor GSK343 reduced viability and proliferation in both dGSCs and parental cells, independent of H3K27me3 modulation, as a global methylation inhibitor exerted no effect. Mechanistically, GSK343 triggered ferroptosis, driven by increased NRF2, lipid peroxidation, and intracellular iron release, rather than apoptosis, necrosis, or pyroptosis. These findings identify EZH2’s non-canonical, phosphorylation-dependent activity as a key driver of GSC resistance and support EZH2 inhibition as a viable strategy to sensitize resistant GBM cells to ferroptotic cell death pathways [49].

Korleski et al. identified an upstream epigenetic axis driving GSCs formation and proposed a novel nanoparticle-based strategy to target it. The authors demonstrated that the core reprogramming factors Oct4 and Sox2 directly induce EZH2 expression and PRC2-mediated H3K27me3 deposition in GBM neurospheres, silencing a set of core tumor-suppressive genes, including *CAMK2B*, *EGR3*, *NPAS2*, *PCDH8*, *RGS6*, and *TESC*, which are downregulated in clinical GBM and correlate with poor patient survival. This repression is linked to DNA methylation-dependent silencing of miR-217-5p, a microRNA that directly targets the EZH2 3′UTR. Loss of miR-217-5p in GSCs relieves EZH2 suppression. Conversely, its restoration reactivates PRC2-target genes, reduces stem cell frequency, impairs self-renewal, and suppresses proliferation without affecting baseline viability. Translating these findings in vivo, the authors used bioreducible polymeric nanoparticles (“nanomiRs”) to deliver miR-217-5p mimics directly into orthotopic GBM xenografts. This approach successfully reduced EZH2/H3K27me3 levels, decreased tumor burden, and enhanced sensitivity to ionizing radiation compared with radiation alone. Overall, this work demonstrates that miRNA-mediated disruption of the PRC2/EZH2 axis is a viable, brain-penetrant alternative to small-molecule EZH2 inhibitors (which frequently suffer from poor CNS bioavailability), adding miR-217-5p to a growing list of GSC-regulating miRNAs (alongside miR-148a, miR-10b-5p, and miR-296-5p) with clear potential for combinatorial epigenetic and radiosensitizing GBM therapy [151]. The summary of described EZH2 inhibitors is presented in Table 3 below.

## 7. Discussion

The recognition that aberrant histone methylation is a fundamental hallmark of cancer has driven the development of histone methyltransferases and demethylases as novel therapeutic targets [53,55,120]. In GBM, EZH2 has emerged as a key oncogenic regulator that promotes tumor plasticity, GSC maintenance, proliferation, invasion, and resistance to conventional therapies. By reshaping the epigenetic landscape and controlling transcriptional programs that sustain malignancy, EZH2 represents a particularly attractive target for epigenetic intervention [91,97].

Even though numerous studies have implicated EZH2 in glioma progression and GSC biology, the absence of a significant association between EZH2 expression and overall survival in the TCGA-GBM cohort suggests that its biological importance may not be fully reflected by bulk transcriptomic survival analyses. One possible explanation is that GBM represents a uniformly aggressive disease with inherently poor prognosis, limiting the ability of individual molecular markers to further stratify patient outcomes. This is consistent with reports that established GBM molecular subtypes also show no significant association with overall survival in the TCGA-GBM cohort, suggesting that bulk transcriptomic stratification approaches broadly face similar limitations in this disease [152]. In contrast, analyses encompassing gliomas of different grades often reveal stronger prognostic associations because they capture broader differences in tumor biology. Additionally, GBM exhibits marked intratumoral heterogeneity, comprising diverse cellular populations, including stem-like, differentiated, immune, and stromal cells [12]. Since EZH2 has been linked particularly to the maintenance and self-renewal of GSCs [62,69,127], its functional significance may be restricted to a relatively small but biologically critical cellular compartment that is not adequately represented by bulk tumor mRNA measurements. Furthermore, accumulating experimental evidence indicates that EZH2 contributes to resistance to radiotherapy and TMZ as well as to tumor recurrence, suggesting that its principal role may involve modulation of therapeutic response rather than direct determination of baseline patient survival. Consequently, the lack of a prognostic association in GBM does not necessarily preclude EZH2 from being a biologically relevant or therapeutically actionable target, as its impact may depend on specific cellular contexts and treatment-related mechanisms that are not captured by conventional overall survival analyses.

Although direct investigation of a KDM1A–EZH2 signaling axis in GBM remains limited, growing evidence indicates that KDM1A/EZH2 cooperation in GBM is locus- and context-dependent rather than a uniform dual-enzyme mechanism. AGAP2-AS1 exemplifies genuine joint dependence: this lncRNA scaffolds both KDM1A and EZH2 simultaneously at the TFPI2 promoter, where their combined activity silences this tumor suppressor and where disrupting either enzyme alone is sufficient to relieve repression [119]. HOTAIR, by contrast, is capable of binding both PRC2/EZH2 and the KDM1A-CoREST complex [114,116,119] but functions not as a joint scaffold, rather it partitions distinct oncogenic outputs to distinct partners, recruiting EZH2 for cell-cycle control and KDM1A for migratory/mesenchymal phenotypes, with each enzyme independently sufficient for its respective function [103,118]. These observations support a therapeutically relevant but mechanistically heterogeneous KDM1A/EZH2 epigenetic network in GBM: combination inhibition is most directly justified where true dual-enzyme dependence has been demonstrated, as at the AGAP2-AS1/TFPI2 axis, whereas HOTAIR-driven phenotypes may instead call for enzyme-specific targeting matched to the relevant downstream function.

Importantly, no EZH2-targeted therapies have yet received FDA approval for the treatment of GBM. Clinical translation has been hindered largely by the challenge of achieving effective BBB penetration while maintaining sufficient target engagement within the tumor. Nevertheless, multiple preclinical studies indicate that inhibition of EZH2 produces antitumor effects, including suppression of cell-cycle progression, induction of apoptosis, and reduction in stemness-associated phenotypes [113,114,153]. These findings provide a strong rationale for exploring EZH2 targeting strategies in future GBM clinical trials.

EZH2 exhibits biological functions that extend beyond canonical histone modification. In addition to catalyzing H3K27 trimethylation, EZH2 can methylate several non-histone proteins and regulate transcription through methyltransferase-independent mechanisms [44,52,56]. Given its established role in promoting stemness, invasion, and therapeutic resistance in GBM, considerable efforts have focused on the development of EZH2 inhibitors.

Prominent examples include GSK126 and GSK343, which suppress GBM growth and self-renewal in preclinical models [49,69,127,128]. Other agents such as DZNep have also demonstrated anti-glioma activity through modulation of additional signaling pathways, including STAT3 [149,150,154].

However, the successful clinical translation of EZH2-targeted therapies in GBM remains challenging because effective BBB penetration is essential for achieving therapeutic concentrations within the CNS [155]. Using in vitro transport assays and transporter-knockout mice, Zhang et al. found all five tested EZH2 inhibitors (EPZ005687, EPZ-6438, UNC1999, GSK343, GSK126) are substrates of the efflux transporters P-glycoprotein (ABCB1) and/or BCRP (ABCG2), which actively pump these drugs out of the brain. Both transporters restricted EPZ005687 and GSK126, while EPZ-6438 was limited mainly by P-gp alone, an effect fully reversible with the inhibitor elacridar. GSK126 also exhibited poor membrane permeability and negligible oral bioavailability (0.2%, rising to only 14.4% in double-knockout mice), rendering it unsuitable for oral use regardless of transporter effects. Overall, EPZ-6438 emerged as the most promising candidate of the series. Still, the authors conclude that ABCB1/ABCG2-mediated efflux likely limits the clinical usefulness of this entire class of EZH2 inhibitors for treating brain tumors [129].

These findings raise an important translational question of whether the therapeutic potential of EZH2 inhibition in GBM can be fully realized using conventional inhibitors with limited CNS exposure. Although compounds such as GSK126 and GSK343 have demonstrated potent EZH2 inhibition and antitumor activity in preclinical settings, inadequate CNS penetration may restrict their utility in GBM [69,128]. In contrast, the development of next-generation EZH2 inhibitors, such as IHMT-337, suggests that improved BBB permeability and intracranial activity may be achievable through optimized molecular design [71].

Beyond the development of BBB-permeable inhibitors, additional strategies aimed at enhancing drug delivery may further improve the therapeutic potential of EZH2 inhibition. Nanoformulation-based delivery systems, including polymeric nanoparticles, can improve drug stability and enhance tumor accumulation by enabling tumor-directed delivery or receptor-mediated transport mechanisms [156,157,158,159,160]. In this context, Wang et al. developed a nanoparticle-mediated siRNA delivery system for EZH2 silencing in glioma models, providing proof-of-concept for nanotechnology-enabled epigenetic therapy [156]. Furthermore, targeted protein degradation approaches, including proteolysis-targeting chimeras (PROTACs), represent an alternative strategy for modulating EZH2 activity [126]. However, the successful clinical translation of these strategies will require further optimization of BBB permeability, pharmacokinetic properties, safety profiles, and manufacturing feasibility.

Importantly, increasing evidence suggests that EZH2 inhibition may be most effective when combined with other targeted agents. Combinatorial approaches involving CDK4/6 inhibitors [141], DNA methyltransferase inhibitors [161,162], BET inhibitors [161], and other epigenetic modulators have produced encouraging results in preclinical cancer models, reinforcing the concept that disruption of a single epigenetic regulator may be insufficient to overcome the extensive signaling redundancy characteristic of GBM [163,164,165].

For EZH2-targeted therapies, predictive biomarkers remain less well defined. Candidate stratification markers may include elevated EZH2 expression, increased H3K27me3 levels, transcriptional evidence of PRC2 pathway activation, or molecular subgroups exhibiting greater epigenetic dependency. Although none of these biomarkers has yet been prospectively validated for patient selection in GBM, their incorporation into future translational studies may facilitate the development of biomarker-driven therapeutic strategies targeting the EZH2-KDM1A axis.

As the therapeutic potential of these approaches continues to expand, the long-term safety and tolerability of epigenetic inhibition represent important considerations for the clinical translation of EZH2-targeted therapies in GBM. EZH2 is the catalytic subunit of PRC2 and sustained suppression of PRC2 activity with consequent depletion of H3K27me3 may affect normal hematopoietic and neural progenitor populations [166,167,168]. Scott et al. reported that EZH2 inhibition affected CML stem cells while preserving the survival of normal hematopoietic stem cells, suggesting some degree of selectivity [169]. Similarly, Akiyama et al. showed that the dual EZH1/EZH2 inhibitor valemetostat affected leukemic stem/progenitor cells while producing limited effects on normal CD34+ hematopoietic stem/progenitor cells [170]. Within GBM models, some studies have also reported a degree of selectivity of EZH2-targeted approaches toward malignant glioma cells relative to normal astrocytes [71,128]. However, these findings are predominantly preclinical and do not exclude cumulative or tissue-specific toxicities associated with prolonged treatment. The withdrawal of tazemetostat in March 2026 following the emergence of an increased incidence of secondary hematologic malignancies further underscores the importance of long-term safety monitoring.

These considerations are particularly relevant to dual EZH1/EZH2 inhibition, which achieves more comprehensive suppression of PRC2 activity and may therefore enhance antitumor efficacy but could potentially narrow the therapeutic window [171,172]. Similarly, combined EZH2/KDM1A targeting may enhance antitumor activity while raising concerns regarding tolerability during prolonged treatment [114]. Because both EZH2 and KDM1A regulate epigenetic programs that are also essential for normal cellular homeostasis, their sustained combined inhibition could potentially affect normal proliferative and progenitor cell populations [173,174,175]. Taken together, the systemic and CNS safety of sustained or combined EZH2/KDM1A inhibition remains insufficiently characterized and should be carefully evaluated before these strategies are considered for prolonged clinical use in GBM.

Overall, despite substantial progress in the development of EZH2-targeted therapies, significant challenges remain. This protein possesses complex canonical and non-canonical functions that vary according to cellular context, tumor genotype, metabolic state, and chromatin architecture. Furthermore, emerging evidence suggests that its activities are interconnected through shared epigenetic and lncRNA-mediated regulatory networks. Consequently, future therapeutic strategies targeting the EZH2 will likely require careful molecular characterization and patient stratification. Rather than serving as universal anti-GBM therapies, EZH2 inhibitors may ultimately prove most effective within precision medicine frameworks that identify tumors specifically dependent on these epigenetic vulnerabilities.

## 8. Conclusions

EZH2 has emerged as a promising therapeutic target in GBM owing to its central roles in chromatin remodeling, maintenance of stem-like cellular states, and the regulation of treatment resistance. Analyses of publicly available datasets provide evidence that EZH2 expression is elevated in high-grade gliomas, including GBM, and is frequently associated with unfavorable clinical outcomes when gliomas are evaluated collectively. However, the prognostic significance of EZH2 in GBM specifically remains less clear. This distinction is illustrated by analyses of the TCGA-GBMLGG and TCGA-GBM cohorts, in which significant associations between *EZH2* expression and patient survival are observed in the combined glioma population but are not maintained when GBM cases are analyzed separately. Importantly, the absence of a survival association does not necessarily diminish the therapeutic relevance of a molecular target. EZH2 may play a critical role in tumor maintenance and treatment response without serving as a robust prognostic marker. Consequently, if GBM cells depend on EZH2 activity to sustain proliferation, stemness, invasiveness, or resistance to therapy, EZH2 may remain a therapeutically actionable target despite the lack of correlation between bulk-tumor EZH2 expression and overall survival. Therefore, although *EZH2* expression alone may not represent a reliable prognostic biomarker in GBM, accumulating experimental and clinical evidence supports its continued investigation as a therapeutic target whose contribution to GBM biology may be more accurately reflected by its effects on tumor stemness, therapeutic resistance, and disease recurrence than by overall survival alone.

Among EZH2-targeting agents, tazemetostat was the most clinically advanced compound and received FDA-accelerated approval for epithelioid sarcoma and relapsed/refractory follicular lymphoma. It was also evaluated in early clinical studies that included patients with CNS tumors (e.g., NCT03213665). However, in 2026, tazemetostat was voluntarily withdrawn from all indications and markets after the SYMPHONY-1 trial revealed an increased risk of secondary hematologic malignancies (MDS/AML), leading to discontinuation of all ongoing studies and withdrawal of the FDA approvals.

A major challenge limiting the clinical implementation of EZH2-targeted therapies in GBM is the highly restrictive nature of the BBB, which often prevents adequate drug delivery to tumor tissue. Thus, innovative drug-delivery approaches, such as liposomes, polymeric nanocarriers, exosome-mediated delivery systems, and other BBB-targeting nanoformulations, may enhance drug accumulation within intracranial tumors while minimizing systemic toxicity. Such technologies hold considerable promise for clinical implementation of EZH2-targeting therapeutics.

Furthermore, the molecular heterogeneity of GBM, the plasticity of GSC, and the dynamic interplay between epigenetic pathways contribute to treatment resistance and may reduce the efficacy of single-agent epigenetic therapies. Consequently, future therapeutic strategies will likely need to move beyond isolated inhibition of individual epigenetic regulators. Thus, the rational design of combination therapies integrating EZH2 targeting with other epigenetic drugs should be tested.

Additionally, combination strategies with standard-of-care treatment, immunotherapy, PARP inhibitors, anti-angiogenic agents, or metabolic modulators warrant further investigation for their potential to elicit synergistic antitumor effects. However, the rationale for combined EZH2/KDM1A inhibition itself is not uniform across GBM-relevant targets: as discussed above, AGAP2-AS1 mediates genuine joint dual-enzyme dependence at the TFPI2 locus, where both enzymes are independently required for repression, whereas HOTAIR partitions distinct oncogenic functions to each enzyme separately, recruiting EZH2 for cell-cycle control and KDM1A for migratory and mesenchymal phenotypes. This distinction indicates that combined KDM1A/EZH2 inhibition is not universally justified in GBM and should instead be matched to the specific lncRNA scaffold and downstream phenotype involved, rather than applied as a general therapeutic strategy.

Ultimately, advances in genomic, transcriptomic, and epigenomic profiling are expected to facilitate the identification of predictive biomarkers and patient subgroups most likely to benefit from epigenetic interventions. Future mechanistic, translational, and clinical studies should therefore prioritize biomarker-driven approaches and precision medicine strategies to determine whether targeting the EZH2 epigenetic network can achieve meaningful and durable clinical benefit for patients with GBM. Such efforts may pave the way for personalized epigenetic therapies capable of overcoming the current limitations of GBM treatment.

## Figures and Tables

**Table 1:** Non-coding RNA regulatory networks governing EZH2 in GBM.

Non-Coding RNA	Regulatory Axis/Mechanism	Downstream Target and Phenotype	Reference
miR-9	EZH2 occupies the miR-9 transcriptional start site to transcriptionally repress miR-9, thereby de-repressing the pro-invasive receptor CXCR4.	De-repression of CXCR4 → Invasion	[64]
MEG3	EZH2 represses the tumor-suppressive lncRNA MEG3 via H3K27me3.	Activation of miR-21-3p → Proliferation	[74]
NNT-AS1	NNT-AS1 sequesters miR-582-5p, preventing it from suppressing EZH2.	Elevation of EZH2 → EMT and Invasion	[75]
LINC00963	LINC00963 recruits EZH2 to the p21 promoter.	Silencing of p21 → Cell cycle progression	[77]
KCNQ1OT1	EZH2 silences miR-142-3p, allowing accumulation of the lncRNA KCNQ1OT1, which stabilizes HMGBM3.	Stabilization of HMGBM3 → Proliferation	[78]
SNHG7	SNHG7 binds and sequesters miR-138-5p, preventing it from suppressing its own target, EZH2.	De-repression of EZH2 → Proliferation	[76]
DLGAP1-AS1	DLGAP1-AS1 works as a sponge for miR-1297, sequestering it and preventing it from suppressing its target, EZH2.	De-repression of EZH2 → Proliferation, migration, and invasion	[79]
FOXD2-AS1	FOXD2-AS1 recruits EZH2 to the p53 locus, where H3K27me3 deposition suppresses p53 expression.	Silencing of p53 → Tumor growth	[80]
ANCR	ANCR recruits EZH2 to silence *PTEN*.	Activation of AKT/mTOR → Proliferation, migration, and invasion	[81]
SPRY4-IT1	SPRY4-IT1 sponges tumor-suppressive miR-101-3p, resulting in the de-repression of EZH2, which triggers a robust upregulation of VEGFA.	Activation of VEGFR2/AKT/ERK signalling → Angiogenesis	[82]
miR-137	Direct post-transcriptional targeting of EZH2.	Downregulation of EZH2 → Growth suppression	[83]

EZH2—enhancer of zeste homolog 2, EMT—epithelial–mesenchymal transition, lncRNA—long non-coding RNA.

**Table 2:** HOTAIR and AGAP2-AS1 regulatory networks governing KDM1A and EZH2 in GBM.

Non-Coding RNA	Regulatory Axis/Mechanism	Downstream Target and Phenotype	Reference
HOTAIR	Selectively recruits PRC2/EZH2 via its PRC2-binding domain (independent of KDM1A) to mediate transcriptional repression.	Silencing of p16 and p21 → Cell cycle progression and tumor growth	[118]
HOTAIR	Selectively recruits KDM1A via its KDM1A-binding domain (independent of PRC2), altering KDM1A occupancy at target promoters and shifting H3K27ac.	Shifting promoter occupancy/H3K27ac → Migratory and mesenchymal-like state	[103]
AGAP2-AS1	Simultaneously scaffolds both EZH2 (depositing H3K27me3) and KDM1A (removing H3K4me2) at the *TFPI2* promoter, creating a combined repressive chromatin state.	Silencing of TFPI2 → Proliferation, invasion, and inhibition of apoptosis	[119]

EZH2—enhancer of zeste homolog 2, GBM—glioblastoma, KDM1A—lysine-specific demethylase 1.

**Table 3:** Summary of EZH2 inhibitors.

Target	Model	Intervention	BBB/CNS Exposure of the Inhibitor Alone	Outcome	Reference
EZH2	In vitro: U87, LN229, TJ905, primary GBM cells, GSCs derived from GBM cell lines/patient-derived GBM cells Ex vivo: patient-derived GBM cells In vivo: intracranial and BALB/c nude mouse xenografts	GSK343 (EZH2 inhibitor)	N/D	↓ cell viability, proliferation, tumor growth, migration, invasion, stemness, NF-κB signaling, IL-1β, TNF-α, EMT markers, and GPX expression; ↑ G0/G1 cell-cycle arrest, oxidative stress, lipid peroxidation, intracellular iron accumulation, ferroptosis and animal survival	[49,127,128]
EZH2	In vitro: 15 patient-derived BTIC lines (including BT73 and BT147) In vivo: NOD/SCID flank and intracranial xenografts	UNC1999 (EZH2 inhibitor)	Poor BBB penetration demonstrated in vivo (LC/MS analysis of plasma and brain concentrations)	↓ cell viability and self-renewal; ↑ G1 arrest and autophagy-mediated cell death; Synergistic activity with dexamethasone	[48]
EZH2, EZH2-STAT3-Notch1 axis	In vitro: U87-derved GSCs and patient-derived SHG140s GSCs In vivo: orthotopic intracranial and subcutaneous xenografts	GSK126 or siRNA-mediated knockdown of EZH2 and Notch1	N/D	↓ GSCs proliferation, invasion, sphere formation and stemness markers (CD133, Nestin); ↓ STAT3 and Notch1 signaling; ↓ tumor growth; ↑ animal survival	[69]
EZH2-SLC12A5 axis	In vitro: U87, T98G, LN229 and U251 GBM cell lines; hCMEC/D3 BBB model In vivo: orthotopic mouse xenografts	IHMT-337; EZH2 or KCC2 overexpression/siRNA knockdown	BBB permeability demonstrated in vitro (Hcmec/D3 Transwell model)	Promotes EZH2 degradation; ↓ glioma proliferation and migration; Exhibits in vitro BBB permeability and inhibits tumor progression in vivo	[71]
HOTAIR-EZH2 interaction	In vitro: TBD0220, U87-MG and T98G GBM cells In vivo: mouse xenografts	EPIC-0412 alone or combined with TMZ	N/D	↓ DNA repair pathways and MGMT expression; ↑ cell-cycle arrest, apoptosis and TMZ sensitivity	[138]
HOTAIR-EZH2 interaction	In vitro: U87-MG, LN229, T98G, LN18 and primary TBD0220 GBM cells In vivo: orthotopic TBD0220 and subcutaneous LN18 xenografts	EPIC-0628 alone or combined with TMZ	N/D	↓ HOTAIR-EZH2 interaction, MGMT expression, HR-mediated DNA repair and tumor growth; ↑ ATF3 and p21 expression, G1 cell-cycle arrest, apoptosis and TMZ sensitivity	[137]
PRADX-EZH2 interaction	In vitro: TBD0220, U87-MG and T98G GBM cells In vivo: orthotopic and subcutaneous mouse xenografts	EPIC-0307 alone or combined with TMZ	In vivo efficacy in orthotopic glioma model; Brain exposure demonstrated	↓ DNA repair and MGMT expression; ↑ cell-cycle arrest, apoptosis and TMZ sensitivity	[139]
EZH2	In vitro: A172, U87MG, T98G, MOG-G-CCM, LN405, GOS-3, TMZ-resistant cell lines and patient-derived primary cultures	DZNep (EZH2 inhibitor) combined with panobinostat, TMZ, or APR-246	N/D	Additive or synergistic antitumor activity; ↓ clonogenic survival; ↑ apoptosis; DZNep activity was greater in p53 wild-type cells	[150]
EZH2 + CDK4/6	In vitro: 2D and 3D GBM cultures (GBM03, GBM06, GBM14 and GBM15) Ex vivo: patient-derived organoids In ovo: chorioallantoic membrane (CAM) model	GSK126 combined with abemaciclib	N/D	Synergistic reduction in cell viability; ↓ invasion, tumor growth, stemness, and EMT; disrupted ER-mitochondrial homeostasis	[141]
EZH2 + CDK4/6	In vitro: patient-derived GBM06 and GBM15 GBM cell lines	Tazemetostat combined with abemaciclib	N/D	Synergistic reduction in cell viability; ↑ ROS production and endoplasmic reticulum stress	[141]
EZH2 + HDAC + DRD2	In vitro: multiple tumor cell lines, including GBM (U251, T98G)	EPZ-6438 or PF-06821497 (EZH2 inhibitors) combined with ONC201, with or without vorinostat	N/D	Synergistic reduction in cell viability; ↑ apoptosis and activation of the ISR and DR5 signaling	[147]
EZH2-HSP90	In vitro: patient-derived GBM cells (Pt3, Pt3R) In vivo: Pt3R xenografts in NOD/SCID mice	Dual EZH2-HSP90 inhibitor (Compound 7)	BBB penetration reported; Direct brain exposure not assessed	↓ cell viability and ROS catabolism; ↓ CENP proteins; ↑ M-phase arrest, apoptosis and necrosis; overcomes TMZ resistance	[143]
EZH2	In vitro: patient-derived GSC cultured as neurospheres (GBM1A, GBM1B) and induced GSC lines (A172-Oct4/Sox2) In vivo: orthotopic GSC xenografts in athymic nude mice using GBM1A cells	miR-217-5p NanomiRs alone or combined with ionizing radiation	N/D	Reactivates PRC2-repressed genes; ↓ neurosphere formation and tumor growth; ↑ radiosensitivity through epigenetic reprogramming	[151]

EZH2—enhancer of zeste homolog 2, GBM—glioblastoma, GSCs—glioma stem cells. Arrow pointing downwards (↓) = decreased. Arrow pointing upwards (↑) = increased.

## Data Availability

No new data were created or analyzed in this study. Data sharing is not applicable to this article.
